# Short-term time-restricted feeding improves metabolic rhythms and liver mitochondrial bioenergetic function in high-fat diet-fed mice

**DOI:** 10.1152/function.082.2025

**Published:** 2026-02-02

**Authors:** Jennifer A. Valcin, Telisha Millender-Swain, Jodi R. Paul, Brandon K. Collins, Fatme Ghandour, Sameer Al Diffalha, Jennifer S. Pollock, David M. Pollock, Scott W. Ballinger, Karen L. Gamble, Shannon M. Bailey

**Affiliations:** ^1^Division of Molecular and Cellular Pathology, Department of Pathology, UAB Heersink School of Medicine, University of Alabama at Birmingham, Birmingham, Alabama, United States; ^2^Department of Psychiatry and Behavioral Neurobiology, UAB Heersink School of Medicine, University of Alabama at Birmingham, Birmingham, Alabama, United States; ^3^Division of Anatomic Pathology, Department of Pathology, UAB Heersink School of Medicine, University of Alabama at Birmingham, Birmingham, Alabama, United States; ^4^Section of Cardio-Renal Physiology and Medicine, Division of Nephrology, Department of Medicine, UAB Heersink School of Medicine, University of Alabama at Birmingham, Birmingham, Alabama, United States

**Keywords:** circadian clock, high-fat diet, liver steatosis, mitochondrial bioenergetics, time-restricted feeding

## Abstract

Time-restricted feeding (TRF), which confines food intake to specific time periods without altering nutrient content or reducing calories, has shown promise in improving cardiometabolic health. This study tested whether a 2-wk TRF intervention during the active (dark) period could reverse long-term effects of a high-fat diet (HFD) on liver mitochondrial function, steatosis, and metabolism in mice. Male C57BL/6J mice were fed either a normal-fat diet (NFD, 10% kcal fat) or an HFD (45% kcal fat) ad libitum for 18 wk, followed by 2 wk of active period TRF. Assessments included whole body metabolism, gene expression, histopathology, plasma lipid levels, and mitochondrial bioenergetic function. Chronic HFD feeding abolished the day-night difference in the respiratory exchange ratio (RER), altered 24-h expression rhythms of clock, lipid, and mitochondrial metabolism genes in the liver, and eliminated diurnal variation in liver mitochondrial bioenergetics. TRF partially restored RER rhythmicity without altering body composition or reducing caloric intake in HFD mice. TRF also reset 24-h expression rhythms in clock and several metabolic genes, normalized liver and plasma triglyceride oscillations, and reduced small droplet macrosteatosis in the livers of HFD mice. Importantly, TRF improved liver mitochondrial respiration and reduced circulating levels of mitochondrial transcription factor A, a mitochondrially-derived damage-associated molecule pattern, indicating reduced mitochondrial injury in HFD mice. These findings suggest that TRF can rapidly reverse HFD-induced disruptions in metabolic and mitochondrial function, offering a promising new nonpharmacologic strategy for improving liver health in obesity-related metabolic disease.

## Graphical abstract

**Figure:**
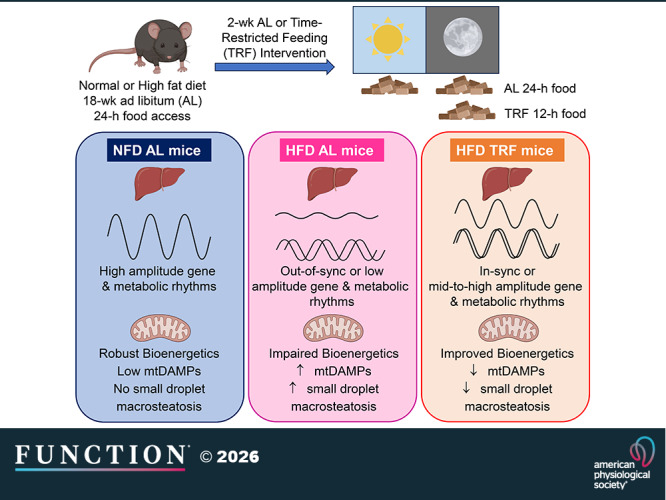

Listen to this article’s corresponding podcast at https://apspublicationspodcast.podbean.com/e/time-restricted-feeding-improves-metabolic-rhythms-and-liver-bioenergetics/.

## INTRODUCTION

Obesity is a risk factor for numerous diseases, including diabetes, hypertension, kidney failure, cognitive decline, and certain cancers. One significant consequence of obesity is fatty liver disease, now referred to as steatotic liver disease (SLD) or metabolic dysfunction-associated steatotic liver disease (MASLD) (1). MASLD is the most common chronic liver disease, affecting up to 35% of the U.S. adult population (2, 3). Managing and treating patients with MASLD primarily involves lifestyle interventions, such as increasing physical activity, adopting healthier diets, and achieving weight loss through these behavioral modifications or through the use of pharmacological means. Notably, two FDA-approved pharmacological therapies, resmetirom (a thyroid hormone β agonist) and semaglutide (a glucagon-like peptide-1 receptor agonist), are now recommended, in combination with lifestyle interventions, for patients with noncirrhotic metabolic dysfunction-associated steatohepatitis (MASH) and fibrosis (4, 5).

Although the quantity (total daily calories) and quality (macronutrient content) of food intake are major contributors to obesity, the timing of eating has also emerged as an important factor. Recent studies indicate that time-restricted feeding (TRF) can offer significant cardiometabolic health benefits in humans (6, 7). Unlike caloric restriction, TRF limits eating to specific times of the day without altering nutrient content or reducing total daily caloric intake. Human studies have demonstrated that TRF improves blood glucose and lipid levels, insulin sensitivity, and blood pressure when eating is confined to daytime hours (6–12). Similarly, in laboratory mice, TRF reduces cardiovascular and metabolic disease risk when feeding is restricted to nighttime hours, i.e., the active period for nocturnal animals (13–15).

TRF may be a promising strategy for improving liver health in patients with MASLD. Clinical studies show that restricting food intake to an 8–10 h window can reduce liver fat and improve liver enzyme profiles, indicating beneficial effects on hepatic function (16–21). In one trial, TRF achieved a 25.8% reduction in liver fat compared to standard care, closely matching a 24.7% reduction observed with calorie restriction during a 16-wk intervention in obese or overweight patients with MASLD (18). These benefits likely result from improved circadian alignment of metabolism, though weight loss and dietary changes in macronutrients may also contribute. Importantly, TRF interventions are generally well tolerated and could complement new pharmacological therapies for MASLD, offering a nondrug approach that targets metabolic dysregulation and aligns with circadian biology.

Multiple pathophysiological factors, including genetic, metabolic, and dietary influences, along with mechanisms such as lipotoxicity, inflammation, and insulin resistance, contribute to the development of MASLD. Patients with severe MASLD often exhibit dysfunctional mitochondria (22–27). Our previous preclinical studies have demonstrated significant changes in liver mitochondrial bioenergetics and the mitochondrial proteome in mice fed a high-fat diet (HFD) with SLD (28–30). Mitochondrial dysfunction likely contributes to liver pathology, as damaged mitochondria cannot provide sufficient energy (ATP) to support cellular repair under HFD conditions (31–33). In addition to causing organ injury, consuming an HFD has been shown to disrupt circadian behaviors, such as food intake and physical activity, in mice (34–36). Interestingly, a growing body of evidence supports the concept that mitochondria exhibit diurnal changes in oxidative metabolism (37, 38) and morphology (39–41). However, our understanding of the effects of HFD and TRF on mitochondrial function is limited. In this study, we aimed to determine whether a brief intervention of active (dark) period TRF could reverse and/or mitigate the long-term effects of an HFD on mitochondrial bioenergetics and liver steatosis.

## MATERIALS AND METHODS

### Animals

Male C57BL/6J mice (6 wk of age; Jackson Laboratory, Bar Harbor, ME) were group-housed (*n* = 4–6 per cage, 20°C–22°C), provided ad libitum (AL) water and standard chow diet, and kept under a 12-h light:12-h dark (12-h LD) schedule where ZT0 = lights-on (07:00 AM) and ZT12 = lights-off (07:00 PM). After 2-wk of acclimation, mice were provided AL access to a high-fat diet (HFD: 45% fat, 4.73 kcal/g, D12451, Research Diets, New Brunswick, NJ) or a normal-fat diet (NFD: 10% fat, 3.85 kcal/g, D12450K, Research Diets, New Brunswick, NJ) and weighed weekly. After 18 wk, half of the cages for each diet group were kept on the AL feeding schedule, and the other half were placed on a time-restricted feeding (TRF) schedule with food provided during the 12-h active (dark) period (ZT12-ZT24) for 2 wk as previously described (15, 42). In the TRF protocol, research personnel removed the food containers at ZT0, emptied them, and returned the empty containers to the cages until ZT12, when food was reintroduced. In the sham protocol, food containers were also removed at ZT0, but they were immediately replaced without being emptied. In both protocols, mice experienced the same handling and container movements. Therefore, the four experimental groups included were NFD AL, HFD AL, NFD TRF, and HFD TRF. Separate cohorts of mice were used for most of the different experimental measurements. Mice were euthanized by exsanguination after isoflurane anesthesia. Gene expression and mitochondrial values reported herein match those of our prior publications where mice were euthanized by decapitation (43, 44). Animal procedures were approved by the UAB IACUC in accordance with the *Guide for the Care and Use of Laboratory Animals* (8th ed., National Academy of Sciences, 2011).

### Whole Body Metabolism

A subset of mice from each of the four diet groups had whole-body fat and lean mass assessed by quantitative magnetic resonance (QMR) imaging (EchoMRI 3-in-1 v2.1; Echo Medical Systems, Houston, TX). After 18 wk of NFD or HFD AL (in standard group housing), mice were transferred to a Comprehensive Lab Animal Monitoring System (CLAMS, Columbus Instruments, Columbus, OH), single-housed, and maintained on AL feeding for one additional week while adjusting to the CLAMS environment (24°C–26°C). At *week 19*, NFD and HFD mice were split into two feeding schedules, AL and TRF, with TRF set to ZT12–ZT24 using the automated CLAMS function. Daily patterns of food intake, physical activity (by beam breaks), respiratory exchange ratio (RER; ratio of carbon dioxide produced to oxygen consumed), and energy expenditure (EE) were recorded and/or calculated for a 2-wk period. CLAMS data from the second week were analyzed using ClockLab software (Actimetrics, Wilmette, IL; 42). Resting metabolic rate (RMR) was determined using EE during the lowest 2-h period in the light period. After CLAMS studies, mice were placed back into standard cages and kept under AL or TRF feeding schedules until a second round of QMR imaging was completed (during *week 22*) for post-TRF whole body analysis.

### RNA Isolation and Real-Time-PCR

Livers were collected at ZT1, 5, 9, 13, 17, and 21, and total RNA was isolated from tissues using TRI-Reagent (Sigma-Aldrich, St. Louis, MO). Isolated RNA was treated with DNA-*free* DNase Treatment and Removal Reagents (Thermo Fisher Scientific, Waltham, MA), and the DNase-treated RNA (260/280 ratio > 1.8) was converted to cDNA using the High-Capacity cDNA Reverse Transcription Kit (Thermo Fisher Scientific, Waltham, MA). Measurements of mRNA levels were done by real-time (RT)-PCR using commercially available gene-specific TaqMan primers from Thermo Fisher Scientific (Waltham, MA) with a QuantStudio6 Flex Real-time PCR system (Thermo Fisher Scientific, Waltham, MA). Determination of mRNA levels was performed using the ΔΔCt method (45) with values normalized to peptidyl-prolyl isomerase A (*Ppia*). Data are presented as a fold-change set to the trough of the control NFD AL mice group (44, 46).

### Liver Measurements and Histopathology

Lipids were extracted from liver tissue, and triglyceride (TG) content (µg TG/mg liver) was measured using L-Type Triglyceride M colorimetric assay reagents (FUJIFILM Wako Diagnostics, Mountain View, CA) by methods described previously (47). Liver histopathology and scoring were conducted as described in our publication (46). Steatosis (% of hepatocytes containing lipid droplets), lobular inflammation (number of inflammatory foci), ballooning, and fibrosis were scored by clinical pathologists blinded to the experimental design using the NAFLD activity scoring (NAS) protocol (48). Steatosis was scored as: 0, <5%; 1, 5%–33%; 2, >34%–65%; and 3, >66% of hepatocytes containing lipid droplets. Lobular inflammation was scored as: 0, no foci; 1, <2 foci; 2, 2–4 foci, and 3, >4 foci. Lobular inflammation and ballooned hepatocytes were rare, and overt fibrosis was not detected; thus, scores reported in Fig. 4*C* and Supplemental Table S7 largely reflect changes in steatosis. Pathologists blinded to the groups also recorded the percentage of hepatocytes containing large and small droplet macrovesicular steatosis (or macrosteatosis).

### Plasma Measurements

Plasma TG (mg/dL) was measured using reagents from Pointe Scientific (Canton, MI; TG reagent, T7532), and nonesterified fatty acids content (NEFA, µM) was measured using Serum/Plasma Fatty Acid Kit (SFA-5, Zen-Bio, Inc, Research Triangle Park, NC). Plasma levels of mitochondrial transcription factor A (mtTFA) were measured using a mouse ELISA Kit (MBS2533527, MyBioSource, Inc., San Diego, CA).

### Mitochondrial Measurements

Fresh liver tissue was homogenized in an ice-cold 250 mM sucrose, 1 mM EDTA, and 5 mM Tris-HCl buffer (pH 7.4), mitochondria were isolated by differential centrifugation methods, and oxygen consumption (i.e., respiration) was measured using an Oxygraph+ system (Hansatech Instruments, PP Systems, Amesbury, MA), as described previously (28, 30, 43, 49). We measured State 3 (St3, ADP-dependent) and State 4 (St4, ADP-independent, post-St3) respiration using Complex I-driven substrates glutamate plus malate (5 mM each) or Complex II-driven substrate succinate (15 mM), in the presence of rotenone (1 μM), with ADP (0.5 mM) added to initiate St3 respiration. We calculated the respiratory control ratio (RCR, St3/St4). Mitochondrial Complex I and Complex IV activities were measured with immunocapture assays (ab109721 and ab109911) based on methods developed by MitoSciences Inc. (Eugene, OR) (50, 51). Mitochondrial Complex V and citrate synthase activities were measured using standard spectrophotometric methods (52). Mitochondrial protein concentration was determined using the bicinchoninic acid protein assay (53; kit 23225, Thermo Fisher Scientific, Waltham, MA).

### Statistical Analysis

We analyzed data using two- or three-factor ANOVA (with or without repeated measures), analysis of covariance (ANCOVA; with lean body mass as a covariate), or *t* tests as appropriate to determine differences among groups. Cosinor analysis was performed to determine whether experimental measures fit a 24-h rhythm. Data were fitted to a cosine wave equation, f(t) = mesor + amplitude × cos[(2πt/T) + acrophase] using a nonlinear regression module (44, 47, 54). The mesor (midline estimating statistic of rhythm) = mean of the rhythm; amplitude = 1/2 the distance between the peak and trough; t = time-point (ZT 1, 5, 9, 13, 17, or 21); T = the period (fixed to 24 h); and acrophase = ZT time of the peak of the rhythm; i.e., cosine maximum. Rhythmicity was determined using a linear regression model f(t) = M + cos (2πt/T) + sin (2πt/T), and data were considered rhythmic if the *P* value of *R^2^* was ≤ 0.05. Student’s *t* test was used to compare parameter estimates among the experimental treatment groups. Data that significantly fit a cosine function (i.e., rhythmic) are represented in graphs by solid lines, whereas lines are not used between time points (i.e., ZTs) when data are nonsignificant (i.e., arrhythmic). Small and large droplet macrosteatosis results were analyzed by ordinal regression. Sample sizes are included in figure legends with statistical significance set at *P* ≤ 0.05. Statistical analyses were done using GraphPad Prism (GraphPad Software Inc., La Jolla, CA) or IBM SPSS Statistics (IBM Corp., Armonk, NY).

## RESULTS

### Whole Body Metabolism

**Figure 1.**
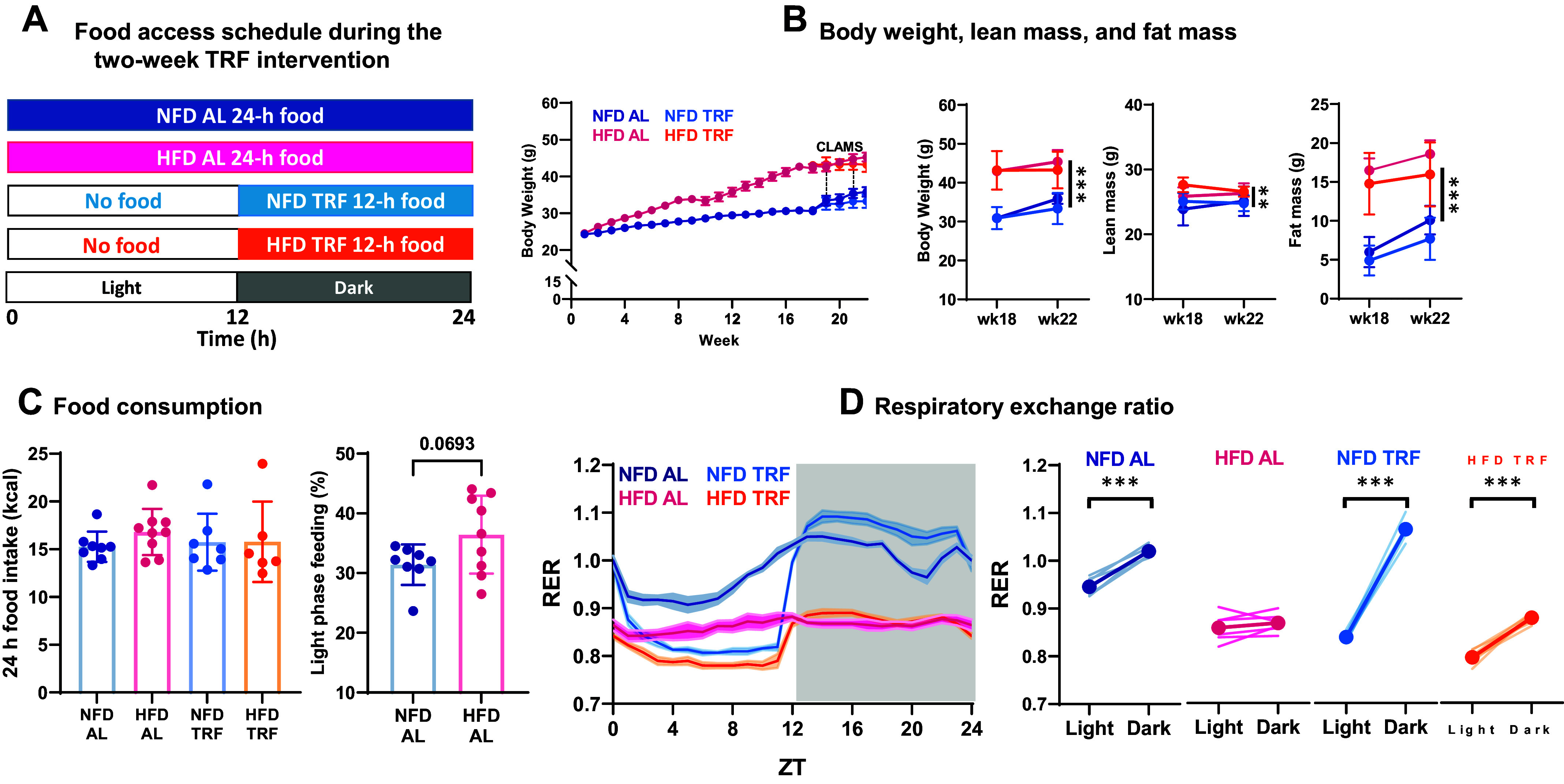
Effect of high-fat diet and time-restricted feeding on whole body metabolic parameters. *A*: diagram illustrating experimental groups and food access schedule during the time-restricted feeding (TRF) intervention – Group 1, NFD AL with 24-h food access (dark blue); Group 2, HFD AL with 24-h food access (pink); Group 3, NFD TRF with 12-h food access (light blue); and Group 4, HFD TRF with 12-h food access (orange). TRF mice were given food only during the 12-h dark period from ZT12 to ZT24. *B*: body weight, lean mass, and fat mass. *Left*: during *weeks 1–18*, there was a significant effect of diet, with HFD mice weighing significantly more than NFD mice (*P* < 0.001, *n* = 11–12 mice per group). *Right*: during week 18, body weight, lean mass, and fat mass were measured using quantitative magnetic resonance (QMR) imaging. Mice were then transferred into a CLAMS for whole body metabolic assessments and subjected to either AL or TRF food access (vertical dashed lines, *left*). Upon completion of CLAMS studies, a second QMR session was done during *week 22* to again measure body weight, lean mass, and fat mass. Before and after TRF via QMR scan: body weight, main effect of diet (*P* < 0.001, *n* = 11–12 mice per group); lean mass, main effect of diet (*P* = 0.01, *n* = 11–12 mice per group); and fat mass, main effect of diet (*P* < 0.001, *n* = 11–12 mice per group). There was no significant diet × food access interactions for all three parameters. *C*: food consumption across the 24-h day for all groups (*left*) and during the 12-h light period of the day for NFD AL and HFD AL groups (*right*). There were no differences in food intake among groups over the 24-h day. HFD AL mice tended to consume a higher percentage of food during the light period than NFD AL mice (*P* = 0.069). *D*: respiratory exchange ratio (RER, ratio of the amount of CO_2_ produced to O_2_ consumed). *Left*: averaged RER traces (*n* = 5 or 6 mice per group). The solid line is the average RER and the shaded region surrounding the line is means ± SE. Gray shaded region indicates the dark period of the day for mice. *Right*, averaged light vs. dark RER (*n* = 5 or 6 mice per group, ***P < 0.001). For three-way ANOVA, all main effects and two-way interactions had *P* < 0.001 except for diet × food access. The interaction of diet × food access × time of day was *P* < 0.001. NFD AL, NFD TRF, and HFD TRF mice have higher RER in the dark period (*P* < 0.001), whereas HFD AL mice do not. Data plotted as mean ± SE. Additional ANOVA results are in Supplemental Tables S1 and S2. AL, ad libitum; HFD, high-fat diet; NFD, normal-fat diet.

Mice were fed either an NFD or an HFD AL for 18 wk, then assigned to AL or TRF feeding schedules (Fig. 1*A*). HFD increased body weight and fat mass compared with NFD mice, while TRF had no effect on body composition or total caloric intake (Fig. 1, *B* and *C*). HFD AL mice consumed more calories during the light phase than NFD AL mice (*P* = 0.069, Fig. 1*C*).

NFD mice displayed a diurnal rhythm in the respiratory exchange ratio (RER) with higher RER in the dark period (Fig. 1*D*). HFD AL mice have an arrhythmic RER (Fig. 1*D*), with TRF partially restoring the day-night difference (Fig. 1*D*). All groups showed day-night differences in energy expenditure (EE; main effect of time, *P* < 0.001), with TRF enhancing this difference (food access × time interaction, *P* < 0.001, Supplemental Fig. S1*A*). HFD increased EE (main effect of diet, *P* = 0.003); however, the effect was greater during the day (*P* < 0.001) than at night (*P* = 0.03). In addition, HFD increased resting metabolic rate (RMR) independent of food access (main effect of diet, *P* < 0.001, Supplemental Fig. S1*C*). Activity was higher at night in all groups, with TRF increasing overall activity and HFD reducing it (Supplemental Fig. S1, *D* and *E*). Statistical results for whole-body metabolic parameters are in Supplemental Tables S1 and S2 or in Supplemental Fig. S1. In summary, 2-wk TRF intervention partially restores the RER rhythm, without altering body weight, fat mass, or daily caloric intake in HFD-fed mice.

### Circadian Molecular Clock Gene Expression

**Figure 2.**
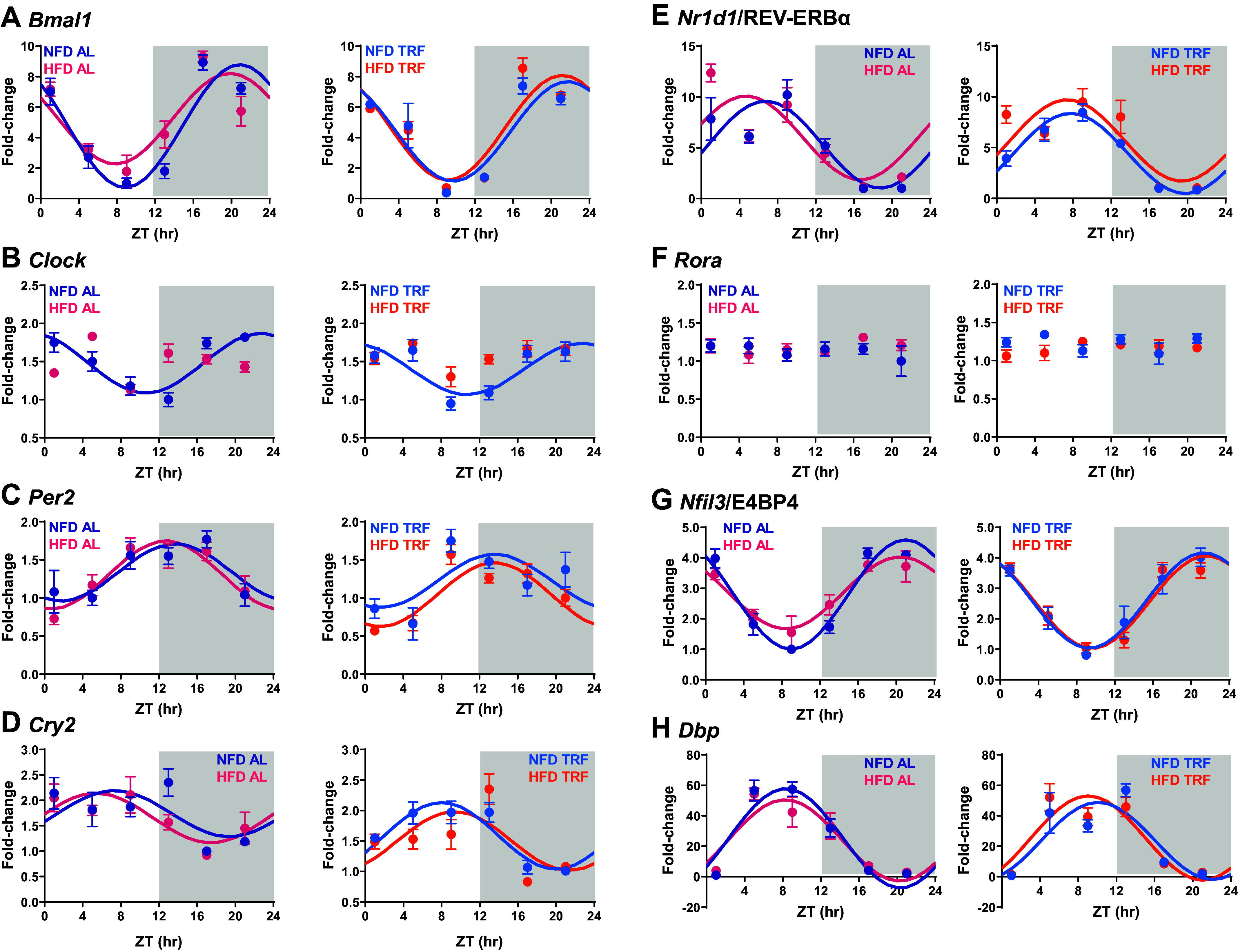
Effect of high-fat diet and time-restricted feeding on 24-h mRNA rhythms of clock genes in the liver. Diurnal mRNA expression of *Bmal1* (*A*), *Clock* (*B*), *Per2* (*C*), *Cry2* (*D*), *Nr1d1*/REV-ERBα (*E*), *Rora* (*F*)*, Nfil3*/E4BP4 (*G*), and *Dbp* (*H*) were measured in liver samples collected from NFD AL (dark blue), HFD AL (pink), NFD TRF (light blue), or HFD TRF (orange) mice every 4 h at ZT1, 5, 9, 13, 17, and 21 (ZT0–ZT12, lights on; ZT12–ZT24 lights off/gray area). Results are presented as mean ± SE for *n* = 4–6 mice per group at each ZT. Cosinor analysis was done using the nonlinear regression module in SPSS. A solid line indicates 24-h rhythmicity and a significant cosine fit (*R*^2^, *P* ≤ 0.05), whereas the absence of a line indicates arrhythmicity and a nonsignificant cosine fit (*R*^2^, *P* > 0.05). Results from cosinor and ANOVA analyses are in Supplemental Tables S3 and S4, respectively. AL, ad libitum; HFD, high-fat diet; NFD, normal-fat diet; TRF, time-restricted feeding.

**Figure 3.**
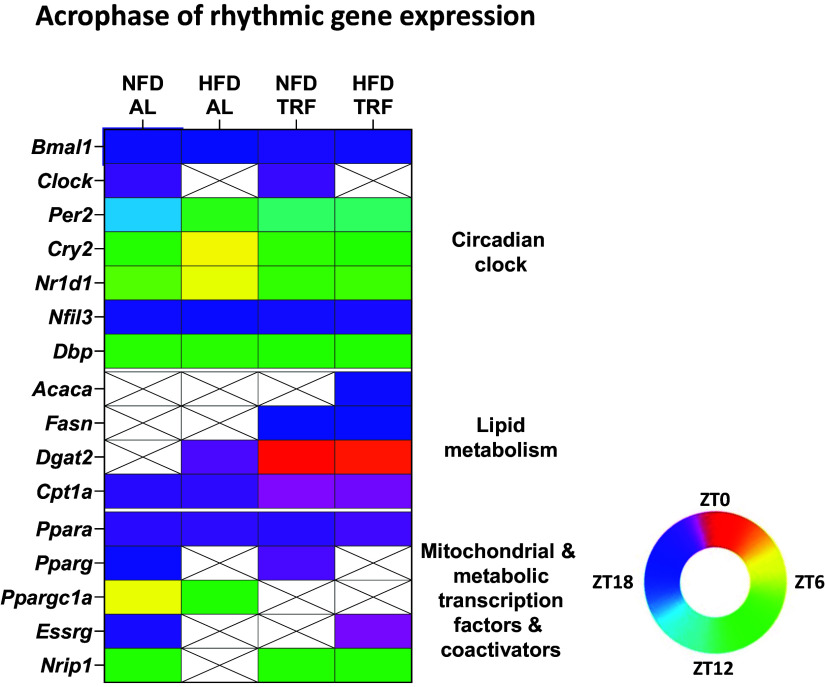
Effect of high-fat diet and time-restricted feeding on the acrophase of 24-h mRNA rhythms in the liver. Heatmap visualizing the acrophase (or time of peak) of the 24-h mRNA rhythm of clock (Fig. 2), lipid metabolism (Fig. 5), and mitochondrial transcription factors and coactivator genes (Fig. 7). Arrhythmic groups are indicated by an X in the cell. Genes where arrhythmicity was present in all groups are not included in the figure.

Cosinor analyses revealed rhythmic expression of most clock genes in the liver (Supplemental Table S3). Basic helix-loop-helix ARNT-like protein 1 (*Bmal1*) expression was rhythmic in all groups (Fig. 2*A*). TRF normalized the mesor and acrophase of *Bmal1* expression in HFD mice (Fig. 2*A*, *right*). Circadian locomotor output cycle kaput (*Clock*) expression was rhythmic in NFD mice, but arrhythmic in HFD mice (Fig. 2*B*). Period 2 (*Per2*) expression rhythm was phased advanced 3.35 h in HFD AL mice compared with NFD AL mice (Fig. 2*C*). Similarly, the cryptochrome 2 (*Cry2*) rhythm was phase advanced 4.04 h in HFD AL compared with NFD AL mice (Fig. 2*D*). TRF significantly decreased the mesor of the *Per2* rhythm in HFD TRF compared with HFD AL mice (Fig. 2*C*). The nuclear receptor subfamily 1 group D member 1 (*Nr1d1,* a.k.a., REV-ERBα) expression rhythm was phase advanced by 2 h in HFD AL compared to NFD AL mice (Fig. 2*E*). Notably, TRF restored the acrophase of the *Cry2* rhythm (Fig. 2*D*) and the *Nr1d1* rhythm (Fig. 2*E*) back to that observed in NFD groups. Retinoic acid receptor-related orphan receptor-alpha (*Rora)* expression was arrhythmic in all groups (Fig. 2*F*). Lastly, nuclear factor, interleukin 3 regulated (*Nfil3*, also known as, E4BP4, Fig. 2*G*) and D-box binding PAR-bZip (*Dbp*, Fig. 2*H*) were rhythmic in all groups. However, the amplitude of the *Nfil3* rhythm was significantly lower in HFD AL compared with NFD AL mice (Fig. 2*G*, *left*). TRF increased the amplitude of the *Nfil3* rhythm in HFD mice back to levels similar to NFD mice (Fig. 2*G*, *right*). TRF delayed the peak of the *Dbp* rhythm in NFD mice (Fig. 2*H*). A heatmap illustrating the acrophase for clock genes with rhythmic expression profiles is provided in Fig. 3. Results from three-factor ANOVA for *Clock* and *Rora* are provided in Supplemental Table S4. Overall, TRF corrected several HFD-induced disruptions in clock gene expression rhythms.

### Liver Histopathology and Plasma Lipids

Liver weight, liver triglyceride (TG) content, and plasma lipids were assessed at 4-h intervals. Cosinor analysis for 24-h rhythms and ANOVA results are included in Supplemental Table S5 and Supplemental Table S6, respectively. A summary of histopathology scores is included in Supplemental Table S7.

**Figure 4.**
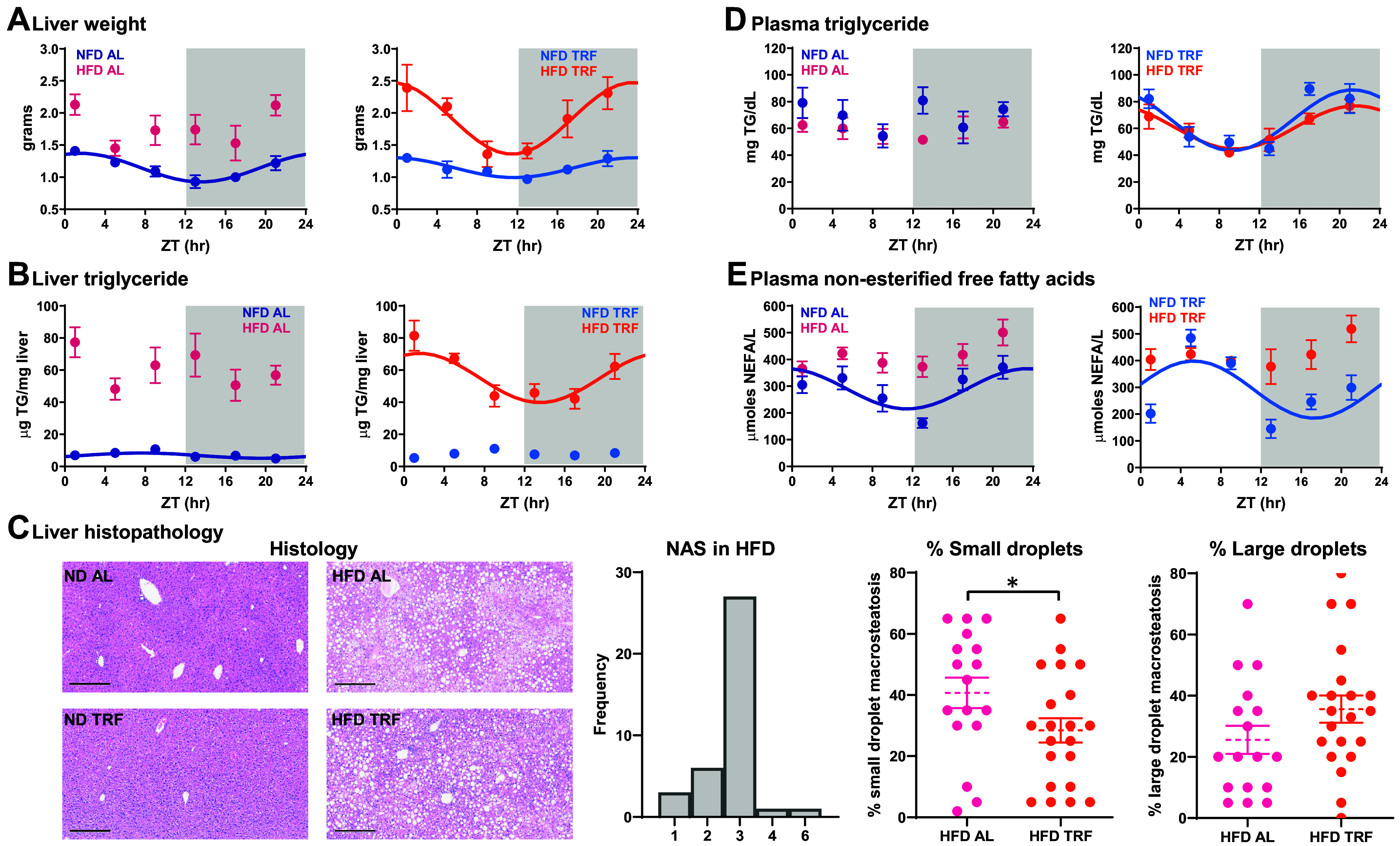
Effect of high-fat diet and time-restricted feeding on 24-h liver and plasma measurements. Liver weight (*A*), liver triglyceride (TG; *B*), histopathology (*C*), plasma TG (*D*), and plasma nonesterified fatty acids (NEFA; *E*) were measured in samples collected from NFD AL (dark blue), HFD AL (pink), NFD TRF (light blue), or HFD TRF (orange) mice at ZT1, 5, 9, 13, 17, and 21 (ZT0–ZT12, lights on and ZT12–ZT24 lights off/gray area). For 24-h data, results are presented as mean ± SE for *n* = 6–12 mice per group at each ZT for liver measures and *n* = 4–6 mice per group at each ZT for plasma measures. Cosinor analysis was done using the nonlinear regression module in SPSS. A solid line indicates 24-h rhythmicity and a significant cosine fit (*R*^2^, *P* ≤ 0.05), whereas the absence of a line indicates arrhythmicity and a nonsignificant cosine fit (*R*^2^, *P* > 0.05). Results from cosinor and ANOVA analyses are in Supplemental Tables S5 and S6, respectively. Results for lipid droplet size were analyzed by ordinal regression. The bar in histopathology panels is 300 µm, and the magnification is ×10. AL, ad libitum; HFD, high-fat diet; NFD, normal-fat diet; TRF, time-restricted feeding.

Liver weight (Fig. 4*A*) and TG (Fig. 4*B*) were higher in HFD mice compared to NFD mice. Both parameters were arrhythmic in HFD AL but rhythmic in HFD TRF mice (Fig. 4, *A* and *B*). Liver TG showed a significant diet × time interaction (*P* = 0.01). Livers from HFD mice have a higher abundance of lipid droplets than those from NFD mice (Fig. 4*C*). There were no time differences in histopathology scoring parameters; therefore, results were combined across light and dark periods. The majority of NFD mice had liver injury scores of 0 (not shown), whereas the majority of HFD mice had liver injury scores of 3 (Fig. 4*C*, NAS). Histopathology scores were driven by steatosis in HFD mice, with minimal inflammation or ballooning (Supplemental Table S7). HFD TRF mice had less small droplet macrosteatosis compared to HFD AL mice [ordinal regression, χ^2^ (1) = 4.155, *P* = 0.042, Fig. 4*C*]. In contrast, large droplet macrosteatosis was more prevalent in HFD TRF mice compared with HFD AL mice, but this difference did not reach statistical significance [ordinal regression, χ^2^ (1) = 2.77, *P* = 0.096, Fig. 4*C*]. Plasma TG was arrhythmic in AL mice and rhythmic in TRF mice (Fig. 4*D*). Plasma TG showed a significant food access × time interaction (*P* = 0.01). Plasma NEFA was rhythmic in NFD mice, but arrhythmic in HFD mice (Fig. 4*E*).

### Lipid Metabolism Gene Expression

**Figure 5.**
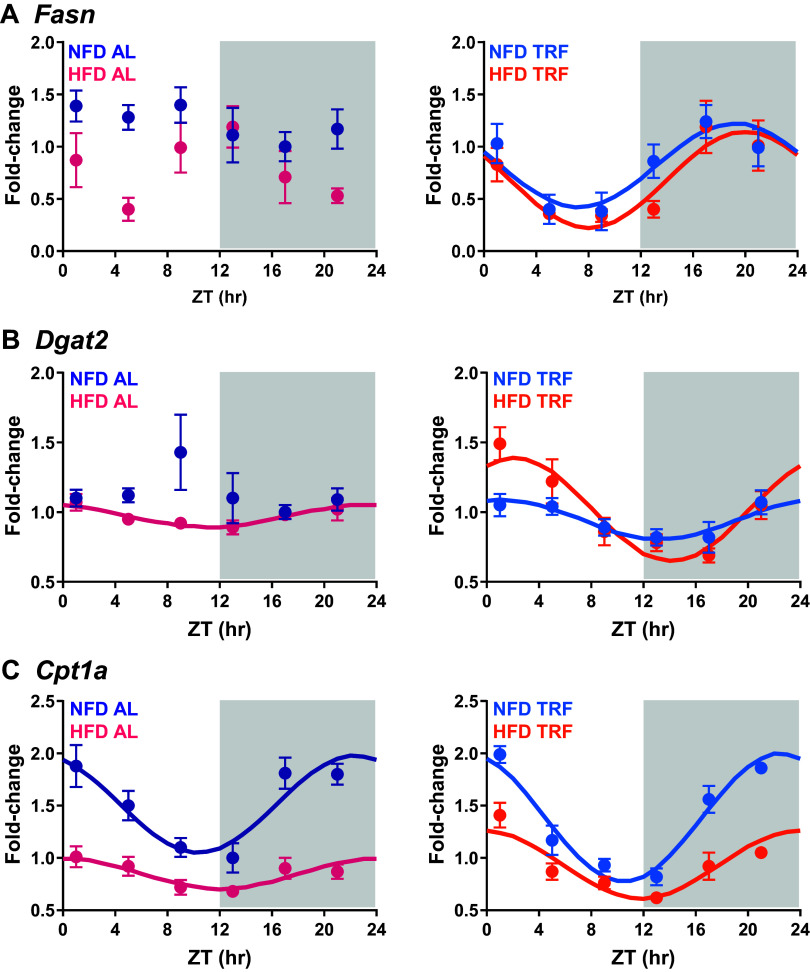
Effect of high-fat diet and time-restricted feeding on 24-h mRNA rhythms of lipid metabolism genes in the liver. Diurnal mRNA expression of *Fasn* (*A*), *Dgat2* (*B*), and *Cpt1a* (*C*) were measured in liver samples collected from NFD AL (dark blue), HFD AL (pink), NFD TRF (light blue), or HFD TRF (orange) mice at ZT1, 5, 9, 13, 17, and 21 (ZT0–ZT12, lights on and ZT12–ZT24 lights off/gray area). Results are presented as mean ± SE for *n* = 4–6 mice per group at each ZT. Cosinor analysis was done using the nonlinear regression module in SPSS. A solid line indicates 24-h rhythmicity and a significant cosine fit (*R*^2^, *P* ≤ 0.05), whereas the absence of a line indicates arrhythmicity and a nonsignificant cosine fit (*R*^2^, *P* > 0.05). Results from cosinor and ANOVA analyses are in Supplemental Tables S8 and S9, respectively. AL, ad libitum; HFD, high-fat diet; NFD, normal-fat diet; TRF, time-restricted feeding.

We next analyzed 24-h expression profiles of select lipid metabolism genes in the liver (Fig. 5). Cosinor analyses for lipid metabolism gene expression are included in Supplemental Table S8. Fatty acid synthase (*Fasn*), the enzyme responsible for synthesizing palmitate from acetyl-CoA and malonyl-CoA, was arrhythmic in AL mice, but rhythmic in TRF mice (Fig. 5*A*). Diacylglycerol O-acyltransferase 2 (*Dgat2*), the enzyme that catalyzes the synthesis of TG, was arrhythmic in NFD AL mice, but had a low amplitude rhythm in HFD AL mice (Fig. 5*B*, *left*). In contrast, *Dgat2* was rhythmic in NFD and HFD TRF mice (Fig. 5*B*, *right*). *Cpt1a*, a mitochondrial protein involved in transferring long-chain fatty acids into the mitochondrial matrix for fatty acid oxidation (FAO) was rhythmic in all groups (Fig. 5*C*). Yet, the mesor and amplitude of *Cpt1a* rhythm was significantly lower in HFD mice compared with NFD mice (Fig. 5*C*) with TRF partially restoring the amplitude of the *Cpt1a* rhythm in HFD mice (Fig. 5*C*, *right*). A heatmap illustrating the acrophase of rhythmic lipid metabolism genes is in Fig. 3. Results for other lipid metabolism genes are shown in Supplemental Fig. S2.

Results from three-factor ANOVA for lipid metabolism gene expression are in Supplemental Table S9. *Fasn* and *Dgat2* showed significant diet × food access and food access × time interactions. HFD AL mice have lower *Fasn* expression compared with NFD AL mice (*P* < 0.0001). *Fasn* expression is also higher in NFD AL mice compared to NFD TRF mice (*P* < 0.0001). Similarly, HFD AL mice have lower *Dgat2* expression compared with NFD AL mice (*P* = 0.005). *Dgat2* expression is higher in NFD AL mice compared with NFD TRF mice (*P* = 0.002).

### Mitochondrial Bioenergetic Function

**Figure 6.**
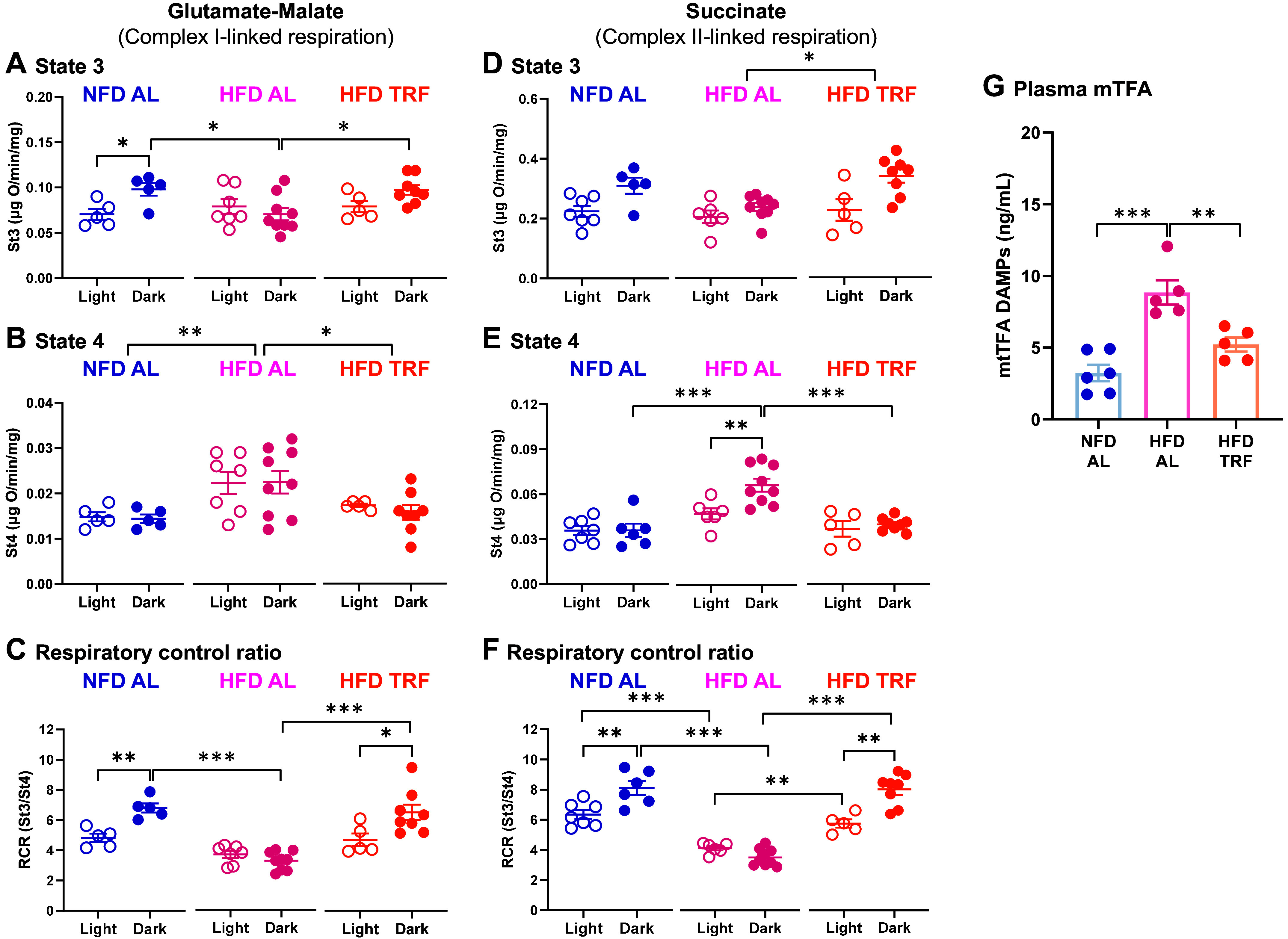
Effect of high-fat diet and time-restricted feeding on mitochondrial respiration in the liver. *A–F*: mitochondrial bioenergetic function was determined in livers from NFD AL (dark blue), HFD AL (pink), and HFD TRF (orange) mice collected during the light (ZT1–ZT3) and dark (ZT13–ZT15) periods of the day. Mitochondrial oxygen consumption was assessed using glutamate plus malate (*left*, *A–C*) and succinate (*right*, *D–F*) as oxidizable substrates for Complex I and Complex II-linked respiration, respectively. State 3 (*A* and *D*) and State 4 (*B* and *E*) respiration was measured in the presence and absence of ADP, respectively, and the respiratory control ratio (RCR, state 3/state 4; *C* and *F*) was calculated. *G*: plasma levels of mitochondrial transcription factor A (mTFA) were measured in pooled samples collected at ZT5–ZT9. Results are presented as mean ± SE and *P* values from post hoc tests are provided in graphs (*P < 0.05, **P < 0.01, ***P < 0.001) with ANOVA results in Supplemental Table S10. AL, ad libitum; HFD, high-fat diet; NFD, normal-fat diet; TRF, time-restricted feeding.

Next, we investigated whether TRF improves liver mitochondrial bioenergetics in HFD mice. Liver mitochondria from NFD AL mice exhibit diurnal variation in Complex I-linked ADP-dependent State 3 (St3) respiration, with higher oxygen consumption during the dark period (Fig. 6*A*). In contrast, mitochondria from HFD AL mice show no day-night difference in St3 respiration (Fig. 6, *A* and *D*). During the dark period, Complex I-linked St3 respiration was reduced in mitochondria from HFD AL mice compared to both NFD AL mice (*P* = 0.03) and HFD TRF mice (*P* = 0.016, Fig. 6*A*). Complex II-linked St3 respiration was significantly higher in HFD TRF mice than in HFD AL mice (*P* = 0.013, Fig. 6*D*).

We also observed elevated ADP-independent St4 respiration in HFD AL mice compared to NFD AL and HFD TRF mice (Fig. 6, *B* and *E*), suggesting increased proton leak or higher oxygen demand to maintain mitochondrial membrane potential (i.e., proton gradient) in HFD AL mice. In addition, mitochondria from NFD AL showed robust diurnal variation in the respiratory control ratio, which was significantly blunted in HFD AL mice but was restored by TRF (Fig. 6, *C* and *F*). Consistent trends were also observed for activities of NADH dehydrogenase (Complex I) and cytochrome *c* oxidase (Complex IV), while ATP Synthase (Complex V) and citrate synthase activities were unaffected by diet, food access, or time of day (Supplemental Fig. S3). Finally, we measured circulating levels of mitochondrial transcription factor A (mtTFA), a mitochondrially-derived damage-associated molecular pattern or mtDAMP (55–57). Plasma mtTFA levels were elevated in HFD AL mice compared with NFD AL mice, but TRF reduced these levels to those seen in NFD AL mice (Fig. 6*G*). ANOVA results for mitochondrial respiration are provided in Supplemental Table S10.

### Mitochondrial Metabolism-Related Transcription Factors and Coactivators

**Figure 7.**
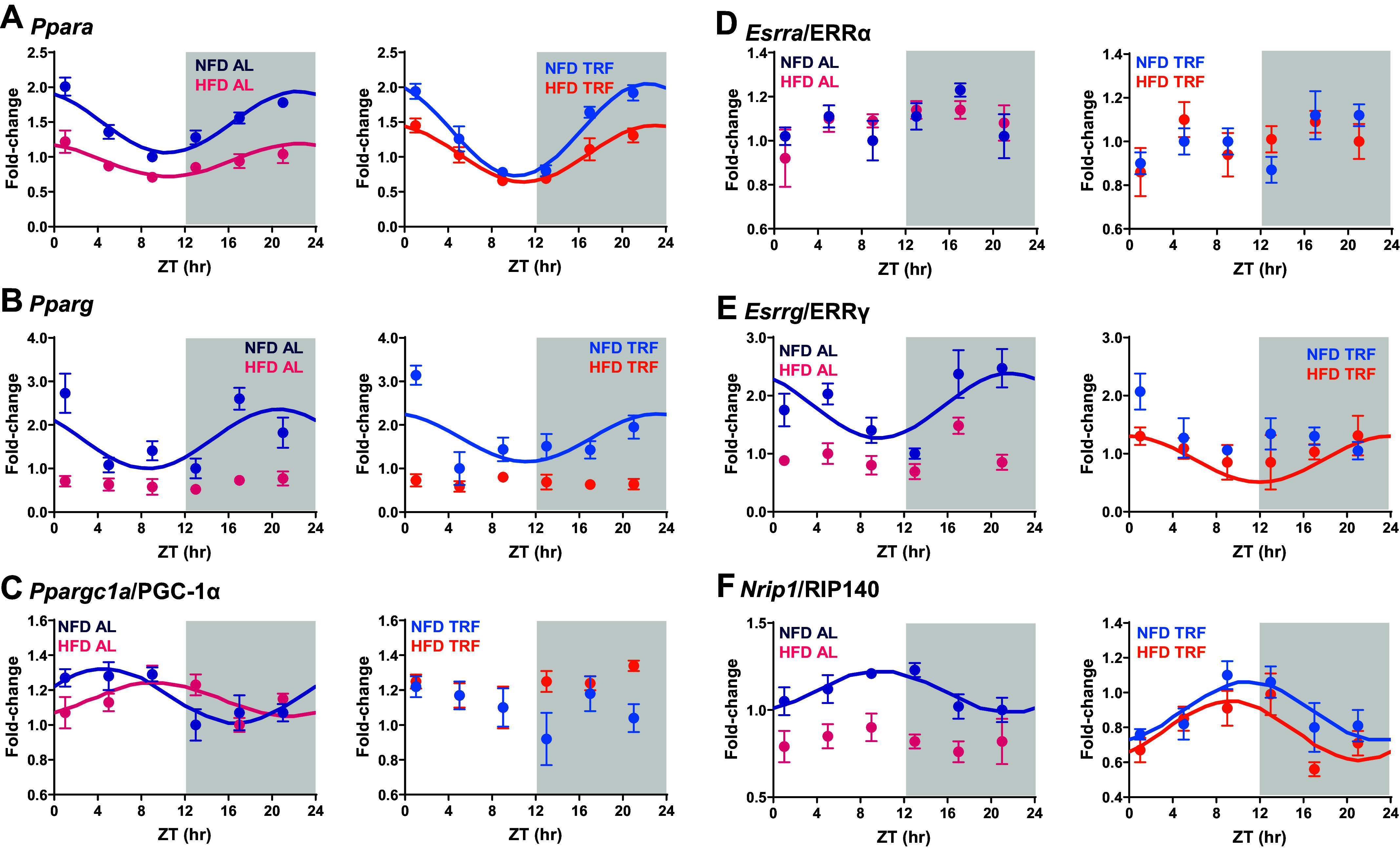
Effect of high-fat diet and time-restricted feeding on 24-h mRNA rhythms of mitochondrial and energy metabolism transcription factors and coactivators in the liver. Diurnal mRNA expression of *Ppara* (*A*), *Pparg* (*B*), *Ppargc1a*/PGC-1α (*C*), *Esrra*/ERRα (*D*), *Esrrg*/ERRγ (*E*), and *Nrip1*/RIP140 (*F*) were measured in liver samples collected from NFD AL (dark blue), HFD AL (pink), NFD TRF (light blue), or HFD TRF (orange) mice at ZT1, 5, 9, 13, 17, and 21 (ZT0–ZT12, lights on and ZT12–ZT24 lights off/gray area). Results are presented as mean ± SE for *n* = 4–6 mice per group at each ZT. Cosinor analysis was done using the nonlinear regression module in SPSS. A solid line indicates 24-h rhythmicity, and a significant cosine fit (*R*^2^, *P* ≤ 0.05), whereas the absence of a line indicates arrhythmicity and a nonsignificant cosine fit (*R*^2^, *P* > 0.05). Results from cosinor and ANOVA analyses are in Supplemental Tables S9 and S11, respectively. AL, ad libitum; HFD, high-fat diet; NFD, normal-fat diet; TRF, time-restricted feeding.

Lastly, we measured 24-h mRNA expression of trans-cription factors and coactivators that regulate lipid and mitochondrial metabolism. Cosinor analysis results for mitochondrial transcriptional regulator genes are included in Supplemental Table S11. Peroxisome proliferator-activated receptor alpha (*Ppara*) was rhythmic in both NFD and HFD groups; however, the mesor and amplitude of the rhythm was lower in HFD AL mice compared with other groups (Fig. 7*A*). Peroxisome proliferator-activated receptor gamma (*Pparg*) was rhythmic in NFD mice, but arrhythmic in HFD mice (Fig. 7*B*). The transcriptional coactivator peroxisome proliferator-activated receptor gamma coactivator 1-alpha (*Ppargc1a*/PGC-1α) was rhythmic in AL mice with the peak of the *Ppargc1a* rhythm occurring 5 h later in HFD AL mice (ZT9.36) compared with NFD AL mice (ZT4.63, Fig. 7*C*, *left*). *Ppargc1a* was arrhythmic in TRF mice (Fig. 7*C*, *right*).

We also measured expression for estrogen-related receptor alpha (*Esrra*/ERRα) and gamma (*Esrrg*/ERRγ) that regulate mitochondrial biogenesis and function (58–62). *Esrra* was arrhythmic in all groups (Fig. 7*D*). *Esrrg* was rhythmic in NFD AL mice, but arrhythmic in HFD AL mice (Fig. 7*E*, *left*). Similarly, nuclear receptor interacting protein 1 (*Nrip1*/RIP140), a transcriptional co-regulator of oxidative metabolism (63–65), was rhythmic in NFD AL mice and arrhythmic in HFD AL mice (Fig. 7*F*, *left*). Critically, TRF reinstated rhythmicity of both *Esrrg* (Fig. 7*E*, *right*) and *Nrip1* (Fig. 7*F*, *right*) in HFD mice. A heatmap illustrating the acrophase for rhythmic mitochondrial regulator genes is provided in Fig. 3.

Three-factor ANOVA results for mitochondrial metabolic gene expression are in Supplemental Table S9. For *Pparg*, the significant three-way interaction (*P* = 0.05) was likely driven by the difference in peaks between NFD groups, given that there were no differences among ZTs or between feeding regimens in the HFD groups (post hoc simple main effects with Sidak correction). For *Esrrg*, the significant three-way interaction (*P* = 0.008) was driven by two differing responses; *1*) *Esrrg* expression was lower in HFD groups, especially in the dark when expression peaks in the NFD AL group, and *2*) minimal time-of-day differences in TRF groups. Although *Esrra* expression was arrhythmic in all groups, a main effect of time (*P* = 0.002), independent of diet and food access, was observed, with an overall increase in mRNA during the dark period. *Esrra* was also lower in TRF mice compared with AL mice (*P* = 0.01). *Nrip1* was higher at the light-dark transition and was not dependent on the diet or food access (*P* < 0.0001). HFD decreased overall *Nrip1* in AL mice but not in TRF mice (diet × food access, *P* = 0.01). *Ppargc1a* was higher in HFD TRF mice compared with NFD TRF mice (*P* = 0.005).

## DISCUSSION

A 2-wk, 12-h active-period TRF intervention improved liver mitochondrial function in HFD-induced obesity. TRF partially restored RER rhythmicity, normalized clock and some lipid metabolism gene expression rhythms, and improved day-night oscillations in mitochondrial respiration alongside restoring rhythmic expression of *Esrrg* and *Nrip1* in HFD-fed mice. Although steatosis remained largely unchanged in HFD mice, TRF reinstated rhythmic TG levels and remodeled lipid droplet morphology. A key strength of this study is its focus on early molecular and metabolic responses, supporting the view that TRF acts at the mitochondrial level before phenotypic changes occur, highlighting a novel mechanism for circadian-driven metabolic repair.

Using CLAMS, we found that feeding mice an HFD (45% kcal fat) in an ad libitum (AL) manner for 20 wk eliminated the day-night difference in the RER compared to mice fed an NFD (10% kcal fat). HFD AL mice also ate a greater percentage of their food during the lights-on, inactive period of the day compared with NFD AL mice. This finding is consistent with prior reports showing that an HFD disrupts normal feeding-fasting behavior (34, 36, 66), including our own collaborative studies examining the effects of HFD and TRF on kidney, vascular, and brain health (15, 42). Here, we confirmed that a brief, 2-wk TRF intervention enhanced RER rhythmicity in mice that continued to eat an HFD. Importantly, this occurred without reductions in body weight, fat mass, and total daily caloric intake. This contrasts with prior studies where HFD animals maintained on long-term TRF feeding protocols (e.g., >12 wk) had lower body weights and less fat mass compared with AL groups (13, 67–69). Therefore, our study design is advantageous as it allows us to test whether TRF can lessen HFD-induced molecular, biochemical, and/or functional impairments in the liver before affecting body weight and/or adiposity.

Our results align with several observations indicating that an HFD disrupts regular diurnal expression patterns of circadian clock genes in the liver (69–73). For instance, Kohsaka et al. (35) demonstrated that the diurnal gene expression profiles of core clock genes such as *Bmal1, Clock,* and *Per2* were altered in the livers of mice fed an HFD (45% kcal fat) for 6 wk. Building on this, Hatori et al. (68) showed that long-term TRF (an 8-h feeding window during the active period for over 16 wk) prevented HFD-induced disruptions in clock gene expression rhythms and metabolic impairments in the liver. These findings have been corroborated in other studies (13, 67, 69, 74). Notably, our brief TRF intervention restored the acrophase (peak time) of *Bmal1, Cry2,* and *Nr1d1* mRNA rhythms in HFD-fed livers to times comparable to those in NFD-fed mice (Supplemental Table S3). Our results demonstrate that reinstating a normal feeding-fasting cycle for just 2 wk is likely sufficient to reestablish nutrient and metabolic signals that entrain the liver molecular clock.

Although the precise signaling mechanisms responsible for normalizing the liver clock remain elusive, Hatori et al. (68) proposed that TRF may normalize molecular clock rhythms through changing nutrient-sensing pathways such as AMPK, CREB, and mTOR, which interact with clock proteins. Interestingly, TRF can alleviate molecular and metabolic impairments of HFD-induced obesity even in mice lacking functional molecular clocks (14). This work suggests that TRF can prevent dysregulation of metabolic pathways by working downstream or independently from the molecular clock mechanism. In support of this idea, other studies indicate that rhythmic food intake may be a more critical regulator of rhythmic gene expression than the molecular clock in multiple organs (75–77). Collectively, these studies highlight the importance of meal timing in regulating metabolism and support the concept that TRF can be an effective strategy for managing metabolic disease, even in the absence of a functional molecular clock. Future studies are needed to determine the contribution of cell-specific clock-dependent and independent mechanisms in TRF.

Our studies demonstrate that even a relatively short-term dietary intervention can positively impact liver metabolism in obese animals. For example, this 2-wk TRF protocol was sufficient to improve rhythmicity in lipid metabolic parameters and gene expression, highlighting its potential to counteract some of the adverse effects of an HFD within a short timeframe. TRF restored rhythmicity in the liver weight and fat content in HFD mice. TRF also induced rhythmicity in circulating TG levels in both NFD and HFD mice, suggesting that setting a regular food intake schedule promotes rhythmicity in lipid metabolism processes. TRF also reinstated or enhanced the expression rhythms of lipid metabolism genes, including *Fasn, Dgat2,* and *Cpt1a*. For example, the amplitude of the *Dgat2* and *Cpt1a* rhythms was significantly higher in the livers of HFD TRF mice compared with HFD AL mice, and TRF normalized the acrophase of the *Dgat2* rhythm to match the peak time observed in NFD TRF mice.

TRF did not improve the overall steatosis grade or histopathology score in the livers of HFD mice, although we observed reduced small droplet and greater large droplet macrosteatosis in HFD mice subjected to TRF. Interestingly, a similar pattern was found in our previous studies, where high levels of small droplet macrosteatosis were observed in mice with a nonfunctional liver clock (i.e., hepatocyte-specific genetic deletion of *Bmal1*) fed an alcohol-containing diet (46). These findings suggest that the liver clock influences lipid droplet morphology, as an HFD or alcohol plus *Bmal1* deletion was associated with shifts toward smaller lipid droplets. This is notable because prior clinical studies report that increased small droplet macrosteatosis and decreased liver ATP levels are linked to worse outcomes in liver transplant patients (78, 79), suggesting the potential pathological relevance of lipid droplet size or distribution in liver health.

An important consideration in interpreting these findings is that mice were housed at standard facility temperatures (∼20°C–26°C) that are below their thermoneutral zone (∼30°C), which increases whole body EE through cold-induced thermogenesis, driving greater substrate oxidation (e.g., fatty acids), and activating brown adipose to maintain body temperature (80–82). These metabolic adaptations could have attenuated liver fat accumulation, potentially reducing the severity of liver injury in our HFD-fed mice. Consequently, the lack of improvement in histopathology scores with a short-term TRF intervention may reflect a baseline reduction in liver fat and pathology due to cold stress rather than an absence of TRF efficacy. Interestingly, prior studies demonstrate that thermoneutral housing worsens liver pathology in HFD-fed mice, including females that are typically resistant to obesity and MASLD (83, 84). This suggests that TRF effects on liver structure and function may be more pronounced under thermoneutral conditions. Future studies conducted at thermoneutrality are therefore needed to more fully evaluate TRF’s therapeutic potential in mitigating diet-induced liver disease.

In our studies, liver mitochondria from NFD AL mice exhibit day-night differences in ADP-dependent St3 respiration and RCR, with higher function during the dark (active) period compared with the light (inactive) period. In contrast, mitochondria from HFD AL mice showed diminished or absent day-night variation in these bioenergetic parameters. The RCR, the ratio of respiration supporting ATP synthesis to that compensating for proton leak, serves as an indicator of mitochondrial efficiency. A lower RCR reflects reduced ATP-generating capacity and impaired efficiency, as observed in livers of HFD AL mice, suggesting bioenergetic stress and increased sensitivity to organ injury. In addition, HFD AL mice exhibited increased St4 respiration, indicative of elevated proton leak, a hallmark of mitochondrial inefficiency that may further exacerbate energy demand under stress. We also observed decreased activities of Complex I and Complex IV in HFD AL mice. Complex I is essential for maintaining cellular redox state (i.e., the NAD^+^/NADH ratio) through NADH re-oxidation, while Complex IV is considered the rate-limiting enzyme of mitochondrial respiration.

Remarkably, just 2 wk of TRF improved mitochondrial bioenergetics by increasing respiration to levels comparable with NFD mice and enhancing day-night variation, despite mice continued HFD consumption and minimal resolution of steatosis (Fig. 4 and Supplemental Fig. S4). Importantly, these rhythms persisted in mitochondria isolated from liver samples collected at different times of day, indicating that diurnal variation is intrinsic to the electron transport chain rather than driven by external factors such as substrate supply. TRF also improved mitochondrial efficiency by reducing proton leak and enhancing RCR rhythmicity, suggesting benefits beyond restoring rhythmicity to include improved energy conservation. It is worth noting that glutamate plus malate or succinate were used as primary substrates in these experiments, which do not fully capture physiological hepatic fuel utilization, particularly fatty acids. Incorporating palmitoylcarnitine plus malate in future studies will provide critical insight on hepatic fatty acid oxidation during TRF.

To investigate mechanisms underpinning the beneficial effects of TRF on liver mitochondrial bioenergetics in HFD mice, we also analyzed 24-h expression rhythms of several mitochondrial metabolism-related transcription factors and coactivators. Surprisingly, *Ppargc1a* expression was arrhythmic in TRF mice, though transcript levels were higher in HFD TRF than HFD AL livers during the dark period. TRF enhanced the *Ppara* rhythm and reinstated rhythmicity for *Esrrg*/ERRγ and *Nrip1*/RIP140, regulators of energy metabolism, that interact with coactivators such as PGC-1α/β (58–60, 65, 85, 86). Interestingly, *Esrrg* and *Nrip1* rhythms were anti-phase in NFD livers, suggesting coordinated control of day-night variation in mitochondrial function. TRF also normalized *Bmal1* and *Rev-erba* rhythms, both implicated in regulating mitochondrial function (37, 39, 87–90). Together, these findings support the hypothesis that the health benefits of TRF in obesity are linked to molecular targets and pathways that bridge the gap between transcription and energy metabolism, improving mitochondrial functional rhythms in the liver and possibly other organs.

Damaged mitochondria release DAMPs, such as mtDNA and mtTFA, which activate TLR9 and RAGE signaling, triggering inflammation and oxidative injury (91, 92, 93–97). When bound to mtDNA, mtTFA promotes cellular uptake by binding to RAGE and triggers TLR9 activation (55–57). Elevated circulating mtDAMPs have been reported in hypertension (95, 98–100) and obesity, including mice fed an HFD (45% fat for 12 wk) and obese MASLD patients (101). Excitingly, TRF reduced plasma mtTFA in HFD mice. It has been proposed that the liver is a major source of circulating mtDAMPs (101). As such, liver-derived mtDAMPs are likely significant mediators of extrahepatic tissue injury in obesity. Supporting this, we have shown that TRF improves blood pressure, reduces aortic stiffness, and decreases kidney damage in HFD mice (15). Future studies should examine how circadian rhythms and liver-derived mtDAMPs (including mtDNA) contribute to interorgan disease cross talk (liver–kidney–vasculature) and multiorgan injury in obesity.

In conclusion, these findings position TRF as a promising, nonpharmacologic strategy to improve mitochondrial function in obesity-related liver disease. Notably, a brief TRF intervention restored or improved key metabolic and molecular rhythms, improved hepatic mitochondrial bioenergetic function, and reduced circulating mtDAMPs. However, the mechanisms underlying TRF’s effects on mitochondrial bioenergetic rhythmicity remain unclear. Future mechanistic research should investigate how TRF interacts with the liver molecular clock and metabolic regulators such as ERRγ and RIP140 to drive these improvements in mitochondrial and liver health.

### Limitations of the Study

This study has several limitations. First, we only used male mice, and given known sex-dependent responses to TRF (13), future studies should include females. Second, because differences at the whole-animal level between NFD AL and NFD TRF groups were minimal, we excluded the NFD TRF group from our mitochondrial analyses to focus resources on groups with more pronounced variations. Future studies that include this group will help to clarify the effects of TRF on bioenergetics in lean, nonobese mice. Third, while we identified novel TRF-mediated metabolic and mitochondrial changes, the underlying mechanisms remain unclear. Investigating lipid droplet processes (e.g., lipolysis and lipophagy) and mitochondrial dynamics (e.g., fusion and fission) could provide deeper mechanistic insight. Fourth, the role of the liver molecular clock in mediating TRF’s benefits was not fully investigated. Addressing these gaps in future studies will strengthen our understanding of TRF’s therapeutic potential for improving metabolic and liver health in obese individuals. Last, we acknowledge that our small indirect calorimetry sample size limited detection of subtle differences in EE (102–104). However, these findings are supported by a larger coordinated research effort by our laboratories, including published studies (15, 42) showing consistent directional effects, strengthening confidence in our conclusions.

## Supplementary Material

Supplemental Tables S1–S11

Supplemental Figs. S1–S4

## Data Availability

Data from this study can be made available from the corresponding author upon reasonable request.
