# Supplementary material for: Short-term time-restricted feeding improves metabolic rhythms and liver mitochondrial bioenergetic function in high-fat diet-fed mice
Source: Function (Oxf). 2026 Feb 2;7(2):e082-2025. doi: 10.1152/function.082.2025 (PMC13249047; doi:10.1152/function.082.2025)
Supplement: Supplemental Tables S1–S11 [file function-2025-082_suppl_tables_allcombined_final.pdf]

Supplemental Table 1. Two-factor analysis of variance for whole-body composition and food intake.

	Diet		Food access		Diet × Food access	
	**F**	**p-value**	**F**	**p-value**	**F**	**p-value**
**QMR whole-body (g)**	**37.9**	< 0.001	2.1	-	0.02	-
**QMR lean (g)**	**5.2**	0.035	0.002	-	0.16	-
**QMR fat (g)**	**52.2**	< 0.001	**4.6**	0.04	0.01	-
**24-h food intake (kcal)**	0.6	-	0.07	-	0.51	-

F-values in bold font correspond to results with statistically significant p values ($p \leq 0.05$). A dash indicates non-statistically significant p values ($p > 0.05$). Diet (normal fat diet v. high fat diet); Food access (ad libitum feeding v. time-restricted feeding).

Supplemental Table 2. Three-factor analysis of variance for whole-body respiratory exchange ratio (RER).

	Diet		Food access		Time		Diet × Food access		Diet × Time		Food access × Time		Diet × Food access × Time	
	**F**	**p-value**	**F**	**p-value**	**F**	**p-value**	**F**	**p-value**	**F**	**p-value**	**F**	**p-value**	**F**	**p-value**
**RER**	**317.2**	< 0.001	**19.2**	< 0.001	**440.8**	< 0.001	0.171	-	**122.7**	< 0.001	**144.2**	< 0.001	**17.95**	< 0.001

F-value in bold font corresponds to result showing statistically significant p value ($p \leq 0.001$). A dash indicates non-statistically significant p values ($p > 0.05$). Diet (normal fat diet v. high fat diet); Food access (ad libitum feeding v. time-restricted feeding); Time (time of day).

Supplemental Table 3. Cosinor analysis of circadian clock gene mRNA rhythms in liver from ad libitum (AL) and time-restricted feeding (TRF) normal fat diet (NFD) and high fat diet (HFD) mice.

			Rhythmicity		Cosinor Parameters					
	Diet	Food access	R^2	p value R^2	Mesor	p value t-test	Amplitude	p value t-test	Acrophase	p value t-test
Bmal1	NFD	AL	**0.75**	< 0.001	4.76		4.04		20.86	
	HFD		**0.49**	< 0.001	5.24	-	2.97	-	19.82	-
	NFD	TRF	**0.64**	< 0.001	4.41		3.27		21.72	
	HFD		**0.67**	< 0.001	4.65	-	3.44	-	21.15	-
	NFD	AL	**0.75**	< 0.001	4.76		4.04		20.86	
	NFD	TRF	**0.64**	< 0.001	4.41	-	3.27	-	21.72	-
	HFD	AL	**0.49**	< 0.001	**5.24**		2.97		**19.82**	
	HFD	TRF	**0.67**	< 0.001	**4.65**	0.04	3.44	-	**21.15**	0.01
Clock	NFD	AL	**0.54**	< 0.001	1.48		0.39		22.63	
	HFD		0.001	0.98	-	-	-	-	-	-
	NFD	TRF	**0.43**	< 0.001	1.41		0.34		22.73	
	HFD		0.09	0.204	-	-	-	-	-	-
	NFD	AL	**0.54**	< 0.001	1.48		0.39		22.63	
	NFD	TRF	**0.43**	< 0.001	1.41	-	0.34	-	22.73	-
Per2	NFD	AL	**0.30**	0.003	1.33		0.37		**15.69**	
	HFD		**0.46**	< 0.001	1.30	-	0.45	-	**12.34**	0.01
	NFD	TRF	**0.24**	0.02	**1.22**		0.35		13.58	
	HFD		**0.56**	< 0.001	**1.04**	0.05	0.42	-	13.53	-
	NFD	AL	**0.30**	0.003	1.33		0.37		15.69	
	NFD	TRF	**0.24**	0.02	1.22	-	0.35	-	13.58	-
	HFD	AL	**0.46**	< 0.001	**1.30**		0.45		12.34	
	HFD	TRF	**0.56**	< 0.001	**1.04**	0.002	0.42	-	13.53	-
Cry2	NFD	AL	**0.20**	0.026	1.74		0.45		**8.49**	
	HFD		**0.27**	0.006	1.65	-	0.49	-	**4.45**	0.02
	NFD	TRF	**0.61**	< 0.001	1.59		0.54		7.99	
	HFD		**0.30**	0.004	1.50	-	0.48	-	9.35	-
	NFD	AL	**0.20**	0.026	1.74		0.45		8.49	
	NFD	TRF	**0.61**	< 0.001	1.59	-	0.54	-	7.99	-
	HFD	AL	**0.27**	0.006	1.65		0.49		**4.45**	
	HFD	TRF	**0.30**	0.004	1.50	-	0.48	-	**9.35**	0.002
***Nr1d1*/ REV-ERBα**	NFD	AL	**0.50**	< 0.001	5.31		4.28		**6.74**	
	HFD		**0.40**	< 0.001	5.97	-	4.13	-	**4.69**	0.005
	NFD	TRF	**0.79**	< 0.001	**4.42**		3.95		7.76	
	HFD		**0.50**	< 0.001	**5.71**	0.03	4.01	-	7.40	-
	NFD	AL	**0.50**	< 0.001	5.31		4.28		6.74	
	NFD	TRF	**0.79**	< 0.001	4.42	-	3.95	-	7.76	-
	HFD	AL	**0.40**	< 0.001	5.97		4.13		**4.69**	
	HFD	TRF	**0.50**	< 0.001	5.71	-	4.01	-	**7.40**	0.01
Rora	NFD	AL	0.01	0.88	-		-		-	
	HFD		0.10	0.19	-	-	-	-	-	-
	NFD	TRF	0.04	0.53	-		-		-	
	HFD		0.10	0.18	-	-	-	-	-	-
***Nfil3*/ E4BP4**	NFD	AL	**0.82**	< 0.001	2.79		**1.79**		20.96	
	HFD		**0.51**	< 0.001	2.85	-	**1.18**	0.02	20.41	-
	NFD	TRF	**0.70**	< 0.001	2.60		1.57		21.31	
	HFD		**0.74**	< 0.001	2.55	-	1.52	-	21.62	-
	NFD	AL	**0.82**	< 0.001	2.79		1.79		20.96	
	NFD	TRF	**0.70**	< 0.001	2.60	-	1.57	-	21.31	-
	HFD	AL	**0.51**	< 0.001	2.85		1.18		20.41	
	HFD	TRF	**0.74**	< 0.001	2.55	-	1.52	-	21.62	-
Dbp	NFD	AL	**0.79**	< 0.001	25.21		32.58		8.33	
	HFD		**0.61**	< 0.001	23.86	-	26.65	-	8.25	-
	NFD	TRF	**0.58**	< 0.001	23.51		25.29		10.08	
	HFD		**0.63**	< 0.001	25.37	-	27.64	-	9.04	-

NFD	AL	**0.79**	< 0.001	25.21		32.58		**8.33**	
NFD	TRF	**0.58**	< 0.001	23.51	-	25.29	-	**10.08**	0.02
HFD	AL	**0.61**	< 0.001	23.86		26.65		8.25	
HFD	TRF	**0.63**	< 0.001	25.37	-	27.64	-	9.04	-

For rhythmicity, R^2 values in bold correspond to results with statistically significant p values ($p \leq 0.05$). Cosinor parameters in bold correspond to results with statistically significant p values ($p \leq 0.05$), whereas a dash indicates non-statistically significant p values ($p > 0.05$) from Student's t-test.

Supplemental Table 4. Three-factor analysis of variance for *Clock* and *Rora* mRNA expression.

	Diet		Food access		Time		Diet × Food access		Diet × Time		Food access × Time		Diet × Food access × Time	
	**F**	**p-value**	**F**	**p-value**	**F**	**p-value**	**F**	**p-value**	**F**	**p-value**	**F**	**p-value**	**F**	**p-value**
Clock	**4.27**	0.04	0.15	-	**20.17**	< 0.001	**4.22**	0.04	**8.59**	< 0.001	0.10	-	2.27	-
Rora	0.06	-	1.41	-	0.10	-	2.44	-	1.72	-	1.07	-	0.55	-

F-value in bold font corresponds to result showing statistically significant p value ($p \leq 0.05$). A dash indicates non-statistically significant p values ($p > 0.05$). Diet (normal fat diet v. high fat diet); Food access (ad libitum feeding v. time-restricted feeding); Time (time of day)

Supplemental Table 5 Cosinor analysis of liver and plasma measurements from ad libitum (AL) and time-restricted feeding (TRF) normal fat diet (NFD) and high fat diet (HFD) mice.

			Rhythmicity		Cosinor Parameters					
	**Diet**	**Food access**	***R^2***	**p value *R^2***	**Mesor**	**p value t-test**	**Amplitude**	**p value t-test**	**Acrophase**	**p value t-test**
**Liver weight**	NFD	AL	**0.44**	< 0.001	1.15		0.22		1.52	
	HFD		0.09	0.20	-	-	-	-	-	-
	NFD	TRF	**0.28**	0.007	**1.15**		**0.16**		**0.32**	
	HFD		**0.34**	0.001	**1.92**	< 0.001	**0.56**	0.009	**23.47**	< 0.001
	NFD	AL	**0.44**	< 0.001	1.15		0.22		1.52	
	NFD	TRF	**0.28**	0.007	1.15	-	0.16	-	0.32	-
**Liver TG**	NFD	AL	**0.09**	0.045	6.76		1.61		7.53	
	HFD		0.04	0.34	-	-	-	-	-	-
	NFD	TRF	0.01	0.87	-		-		-	
	HFD		**0.21**	0.001	55.13	-	15.33	-	1.53	-
**Plasma TG**	NFD	AL	0.03	0.60	-		-		-	
	HFD		0.47	0.19	-	-	-	-	-	-
	NFD	TRF	**0.10**	< 0.001	66.15		22.73		21.28	
	HFD		**0.40**	< 0.001	60.80	-	16.17	-	21.58	-
**Plasma NEFA**	NFD	AL	**0.24**	0.01	290.25		75.27		23.43	
	HFD		0.04	0.52	-	-	-	-	-	-
	NFD	TRF	**0.29**	0.01	291.78		106.89		5.28	
	HFD		0.07	0.32	-	-	-	-	-	-
	NFD	AL	**0.24**	0.01	290.25		75.27		**23.43**	
	NFD	TRF	**0.29**	0.01	291.78	-	106.89	-	**5.28**	< 0.001

For rhythmicity, R^2 values in bold correspond to results with statistically significant p values ($p \leq 0.05$). Cosinor parameters in bold correspond to results with statistically significant p values ($p \leq 0.05$). A dash indicates non-statistically significant p values ($p > 0.05$) from Student's t-test.

Supplemental Table 6. Three-factor analysis of variance for liver and plasma measurements.

	Diet		Food access		Time		Diet × Food access		Diet × Time		Food access × Time		Diet × Food access × Time	
	**F**	**p-value**	**F**	**p-value**	**F**	**p-value**	**F**	**p-value**	**F**	**p-value**	**F**	**p-value**	**F**	**p-value**
**Liver weight**	**101.12**	< 0.001	0.84	-	**7.08**	< 0.001	0.87	-	1.3	-	1.27	-	1.63	-
**Liver TG**	**427.75**	< 0.001	0.02	-	**2.52**	0.03	0.08	-	**2.93**	0.01	1.35	-	1.08	-
**Plasma TG**	**7.46**	0.007	0.03	-	**6.45**	< 0.001	0.55	-	0.41	-	**2.98**	0.01	1.61	-
**Plasma NEFA**	**56.74**	< 0.001	0.44	-	**8.69**	< 0.001	0.22	-	**3.35**	0.007	1.71	-	**2.56**	0.03

F-values in bold font correspond to results showing statistically significant p values ($p \leq 0.05$). A dash indicates non-statistically significant p values ($p > 0.05$). TG, triglyceride; NEFA, non-esterified fatty acids. Diet (normal fat diet v. high fat diet); Food access (ad libitum feeding v. time-restricted feeding); Time (time of day).

Supplemental Table 7. Liver Histopathology Scores.

**Histology Variable**	**NFD AL** (*N*=14)	**HFD AL** (*N*=17)	**NFD TRF** (*N*=12)	**HFD TRF** (*N*=21)
**NAS**				
0	12 (86)[a]	0 (0)	10 (84)	0 (0)
1	2 (14)	1 (6)	1 (8)	2 (9)
2	0 (0)	2 (12)	0 (0)	4 (19)
3	0 (0)	13 (76)	0 (0)	14 (67)
4	0 (0)	1 (6)	1 (8)	0 (0)
5	0 (0)	0 (0)	0 (0)	0 (0)
6	0 (0)	0 (0)	0 (0)	1 (5)
**Steatosis grade**				
0 - <5%	12 (86)	0 (0)	10 (84)	0 (0)
1 - 5-33%	2 (14)	1 (6)	2 (16)	2 (9)
2 - 34-66%	0 (0)	2 (12)	0 (0)	5 (24)
3 - >66%	0 (0)	14 (82)	0 (0)	14 (67)
**Lobular inflammation**				
0 - none	14 (100)	17 (100)	11 (92)	19 (90)
1 - <2	0 (0)	0 (0)	0 (0)	1 (5)
2 - 2-4	0 (0)	0 (0)	0 (0)	1 (5)
3 - >4	0 (0)	0 (0)	1 (8)	0 (0)
**Ballooning**				
0 - none	14 (100)	16 (94)	12 (100)	19 (99)
1 - few	0 (0)	1 (6)	0 (0)	1 (5)
2 - many	0 (0)	0 (0)	0 (0)	1 (5)

[a]Values are *N* (%).

Supplemental Table 8. Cosinor analysis of lipid metabolism gene mRNA expression in liver from ad libitum (AL) and time-restricted feeding (TRF) normal fat diet (NFD) and high fat diet (HFD) mice.

			Rhythmicity		Cosinor Parameters					
	**Diet**	**Food access**	R^2	**p value** R^2	**Mesor**	**p value t-test**	**Amplitude**	**p value t-test**	**Acrophase**	**p value t-test**
***Acaca*/ ACC1**	NFD	AL	0.10	0.20	-		-		-	
	HFD		0.10	0.12	-	-	-	-	-	-
	NFD	TRF	0.15	0.22	-		-		-	
	HFD		**0.19**	0.03	0.85	-	0.21	-	20.70	-
***Acacb*/ ACC2**	NFD	AL	0.06	0.39	-		-		-	
	HFD		0.07	0.29	-	-	-	-	-	-
	NFD	TRF	0.07	0.38	-		-		-	
	HFD		0.10	0.19	-	-	-	-	-	-
Fasn	NFD	AL	0.12	0.18	-		-		-	
	HFD		0.10	0.18	-	-	-	-	-	-
	NFD	TRF	**0.35**	0.003	0.82		0.40		19.23	
	HFD		**0.40**	< 0.001	0.68	-	0.46	-	20.01	-
Dgat2	NFD	AL	0.08	0.26	-		-		-	
	HFD		**0.18**	0.04	0.97	-	0.08	-	23.16	-
	NFD	TRF	**0.27**	0.01	0.95		**0.14**		1.24	
	HFD		**0.43**	< 0.001	1.02	-	**0.37**	0.049	2.11	-
	HFD	AL	**0.18**	0.04	0.97		**0.08**		**23.16**	
	HFD	TRF	**0.43**	< 0.001	1.02	-	**0.37**	< 0.001	**2.11**	< 0.001
Cpt1a	NFD	AL	**0.49**	< 0.001	**1.52**		**0.46**		22.37	
	HFD		**0.22**	0.02	**0.85**	< 0.001	**0.15**	0.002	24.05	-
	NFD	TRF	**0.80**	< 0.001	**1.39**		**0.61**		22.47	
	HFD		**0.50**	< 0.001	**0.94**	< 0.001	**0.32**	0.001	23.81	-
	NFD	AL	**0.49**	< 0.001	1.52		0.46		22.37	
	NFD	TRF	**0.80**	< 0.001	1.39	-	0.61	-	22.47	-
	HFD	AL	**0.22**	0.02	0.85		**0.15**		24.05	
	HFD	TRF	**0.50**	< 0.001	0.94	-	**0.32**	0.02	23.81	-
***Srebf1*/ SREBP1c**	NFD	AL	0.043	0.496	-		-		-	
	HFD		0.037	0.538	-	-	-	-	-	-
	NFD	TRF	0.031	0.630	-		-		-	
	HFD		0.051	0.431	-	-	-	-	-	-

For rhythmicity, R^2 values in bold correspond to results with statistically significant p values ($p \leq 0.05$). Cosinor parameters in bold correspond to results with statistically significant p values ($p \leq 0.05$) by Student's t-test. A dash indicates non-statistically significant p values ($p > 0.05$) from Student's t-test.

Supplemental Table 9. Three-factor analysis of variance for metabolic gene expression.

	Diet		Food access		Time		Diet × Food access		Diet × Time		Food access × Time		Diet × Food access × Time	
	F	p-value	F	p-value	F	p-value	F	p-value	F	p-value	F	p-value	F	p-value
Acaca	**12.52**	0.001	1.02	-	0.84	-	0.42	-	0.75	-	1.46	-	1.56	-
Acacb	2.80	-	0.86	-	2.14	-	2.96	-	1.35	-	1.61	-	1.39	-
Dgat2	1.48	-	3.01	-	**5.08**	< 0.001	**7.59**	0.007	2.25	-	**3.55**	0.005	1.30	-
Esrra	0.01	-	**7.15**	0.01	**4.13**	0.002	< 0.01	-	0.72	-	0.78	-	0.81	-
Esrrg	**62.15**	< 0.001	**10.33**	0.002	**7.45**	< 0.001	**6.31**	0.01	0.38	-	**4.26**	0.001	**3.31**	0.008
Fasn	**13.75**	< 0.001	**10.61**	0.002	**2.85**	0.02	**4.20**	0.04	0.34	-	**5.62**	< 0.001	1.57	-
Nrip1	**36.38**	< 0.001	**15.88**	< 0.001	**7.26**	< 0.001	**6.69**	0.01	0.54	-	1.17	-	0.62	-
Ppargc1a	3.00	-	0.07	-	1.35	-	**5.59**	0.02	**4.30**	0.001	**2.90**	0.01	0.34	-
Pparg	**145.71**	< 0.001	0.02	-	**9.26**	< 0.001	0.002	-	**7.10**	< 0.001	**2.58**	0.03	**2.35**	0.05
Srebf1	0.002	-	0.25	-	0.77	-	0.07	-	1.06	-	1.60	-	0.99	-

F-values in bold font correspond to results with statistically significant p values ($p \leq 0.05$). A dash indicates non-statistically significant p values ($p > 0.05$). Diet (normal fat diet v. high fat diet); Food access (ad libitum feeding v. time-restricted feeding); Time (time of day)

Supplemental Table 10. Two-factor analysis of variance for mitochondrial respiration.

	Diet group		Time of day		Diet group × Time of day	
	**F**	**p-value**	**F**	**p-value**	**F**	**p-value**
**Glutamate-malate**						
**State 3**	1.75	-	**4.57**	0.04	**3.82**	0.032
**State 4**	**8.15**	0.0013	0.13	-	0.10	-
**Respiratory control ratio**	**26.93**	< 0.001	**14.2**	< 0.001	**7.55**	0.002
**Succinate**						
**State 3**	**3.64**	0.04	**17.81**	< 0.001	1.73	-
**State 4**	**20.37**	< 0.001	**5.59**	0.024	**3.76**	0.033
**Respiratory control ratio**	**75.83**	< 0.001	**20.32**	< 0.001	**12.86**	< 0.001

F-values in bold font correspond to results with statistically significant p values ($p \leq 0.05$). A dash indicates non-statistically significant p values ($p > 0.05$). Time of day compares light v. dark period.

Supplemental Table 11. Cosinor analysis of transcription factor and coactivator gene mRNA expression in liver from ad libitum (AL) and time-restricted feeding (TRF) normal fat diet (NFD) and high fat diet (HFD) mice.

			**Rhythmicity**		**Cosinor Parameters**					
	**Diet**	**Food access**	R^2	**p value** R^2	**Mesor**	**p value t-test**	**Amplitude**	**p value t-test**	**Acrophase**	**p value t-test**
Ppara	NFD	AL	**0.65**	< 0.001	**1.50**		**0.44**		22.39	
	HFD		**0.35**	0.001	**0.96**	< 0.001	**0.23**	0.02	22.46	-
	NFD	TRF	**0.79**	< 0.001	**1.39**		**0.66**		22.27	
	HFD		**0.58**	< 0.001	**1.05**	< 0.001	**0.41**	0.01	22.95	-
	NFD	AL	**0.65**	< 0.001	1.50		**0.44**		22.39	
	NFD	TRF	**0.79**	< 0.001	1.39	-	**0.66**	0.01	22.27	-
	HFD	AL	**0.35**	0.001	0.96		**0.23**		22.46	
	HFD	TRF	**0.58**	< 0.001	1.05	-	**0.41**	0.05	22.95	-
Pparg	NFD	AL	**0.33**	0.005	1.68		0.68		20.57	
	HFD		0.03	0.026	-	-	-	-	-	-
	NFD	TRF	**0.23**	0.026	1.70		0.55		23.13	
	HFD		0.01	0.843	-	-	-	-	-	-
	NFD	AL	**0.33**	0.005	1.68		0.68		20.57	
	NFD	TRF	**0.23**	0.026	1.70	-	0.55	-	23.13	-
***Ppargc1a*/ PGC-1α**	NFD	AL	**0.30**	0.004	1.17		0.16		**4.63**	
	HFD		**0.19**	0.032	1.15	-	0.09	-	**9.36**	0.01
	NFD	TRF	0.08	0.299	-		-		-	
	HFD		0.17	0.053	-	-	-	-	-	-
***Esrra*/ ERRα**	NFD	AL	0.085	0.242	-		-		-	
	HFD		0.101	0.172	-	-	-	-	-	-
	NFD	TRF	0.091	0.252	-		-		-	
	HFD		0.021	0.713	-	-	-	-	-	-
***Esrrg*/ ERRγ**	NFD	AL	**0.25**	0.011	1.83		0.56		21.67	
	HFD		0.07	0.284	-	-	-	-	-	-
	NFD	TRF	0.09	0.268	-		-		-	
	HFD		**0.44**	< 0.001	0.90	-	0.39	-	23.93	-
***Nrip1*/ RIP140**	NFD	AL	**0.27**	0.007	1.11		0.12		9.71	
	HFD		0.04	0.548	-	-	-	-	-	-
	NFD	TRF	**0.27**	0.011	**0.89**		0.17		10.89	
	HFD		**0.26**	0.008	**0.78**	0.03	0.17		9.20	-
	NFD	AL	**0.27**	0.007	**1.11**		0.12		9.71	
	NFD	TRF	**0.27**	0.008	**0.89**	< 0.001	0.17	-	10.89	-

For rhythmicity, R^2 values in bold correspond to results with statistically significant p values ($p \leq 0.05$). Cosinor parameters in bold correspond to results with statistically significant p values ($p \leq 0.05$) from Student's t-test. A dash indicates non-statistically significant p values ($p > 0.05$) from Student's t-test.
