# Supplementary material for: Short-term time-restricted feeding improves metabolic rhythms and liver mitochondrial bioenergetic function in high-fat diet-fed mice
Source: Function (Oxf). 2026 Feb 2;7(2):e082-2025. doi: 10.1152/function.082.2025 (PMC13249047; doi:10.1152/function.082.2025)
Supplement: Supplemental Figs. S1–S4 [file function-2025-082_suppl_figures_1_4_combined.pdf]

A. Energy expenditure – 12-h

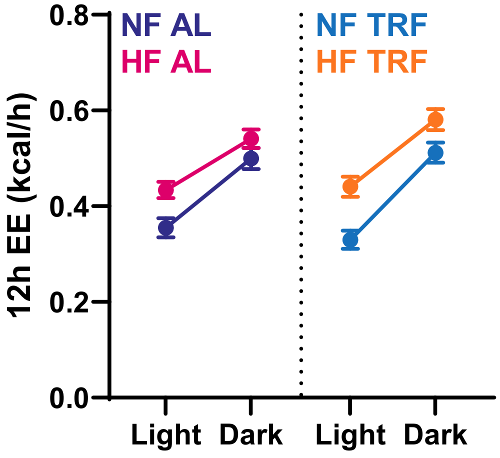

ANCOVA 12-h EE	F	p value	n_p^2
Diet group	11.6	0.003	0.39
Food access	0.2	-	0.01
Time	795.3	< 0.001	0.98
DG × FA	0.7	-	0.04
DG × T	7.8	0.012	0.29
FA × T	17.9	< 0.001	0.49
DG × FA × T	0.0	-	0.00
QMRlean	1.4	-	0.07

B. Energy expenditure – 24-h

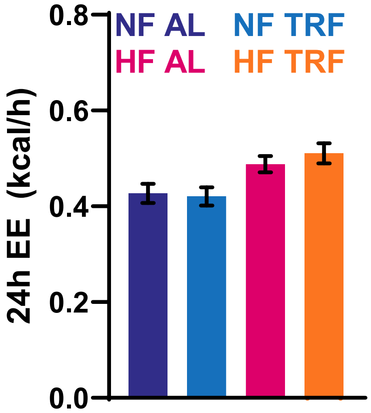

ANCOVA 24-h EE	F	p value	n_p^2
Diet group	11.6	0.003	0.39
Food access	0.2	-	0.01
DG × FA	0.7	-	0.04
QMRlean	1.4	-	0.07

C. Resting metabolic rate – 24-h

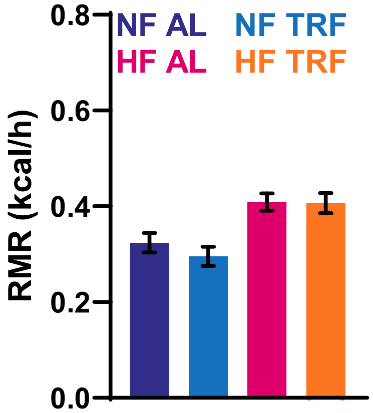

ANCOVA 24-h EE	F	p value	n_p^2
Diet group	19.1	< 0.001	0.52
Food access	0.6	-	0.03
DG × FA	0.5	-	0.03
QMRlean	0.4	-	0.02

D. Activity – 24-h

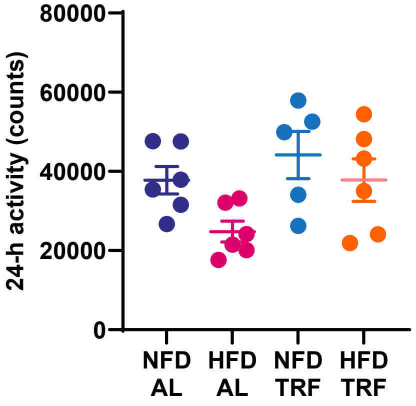

ANOVA	F	p value
Diet group	4.7	0.04
Food access	4.8	0.04
DG × FA	0.57	-

E. Activity – 12-h

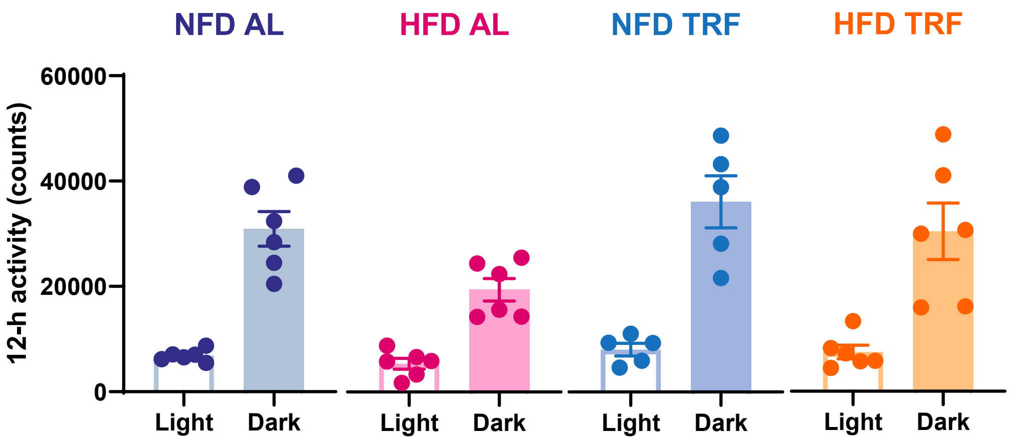

ANOVA	F	p value
Diet group	4.74	0.042
Food access	4.76	0.042
Time	125.9	< 0.001
D × FA	0.57	-
D × T	3.65	-
FA× T	2.61	-
D × FA × T	0.39	-

Supplemental Figure 1. Effect of high fat diet and time-restricted feeding on additional measures of whole-body metabolism and activity. (A) Twelve-hour light and dark period energy expenditure (EE). **(B)** Twenty-four EE. (**C**) Resting metabolic rate (RMR). **(D)** Twenty-four hour and (**E**) 12-h activity levels of mice in the CLAMS. Data presented in panels A, B, and C are displayed as ANCOVA adjusted means $\pm$ SEM, whereas data presented in panels D and E are mean $\pm$ SEM (n = 6 per group). ANCOVA and ANOVA results are provided in tables near bar graphs. F-values in bold font correspond to results with $p \leq 0.05$. A dash indicates $p > 0.05$. Diet (normal fat diet v. high fat diet); Food access (ad libitum feeding v. time-restricted feeding); Time (time of day).

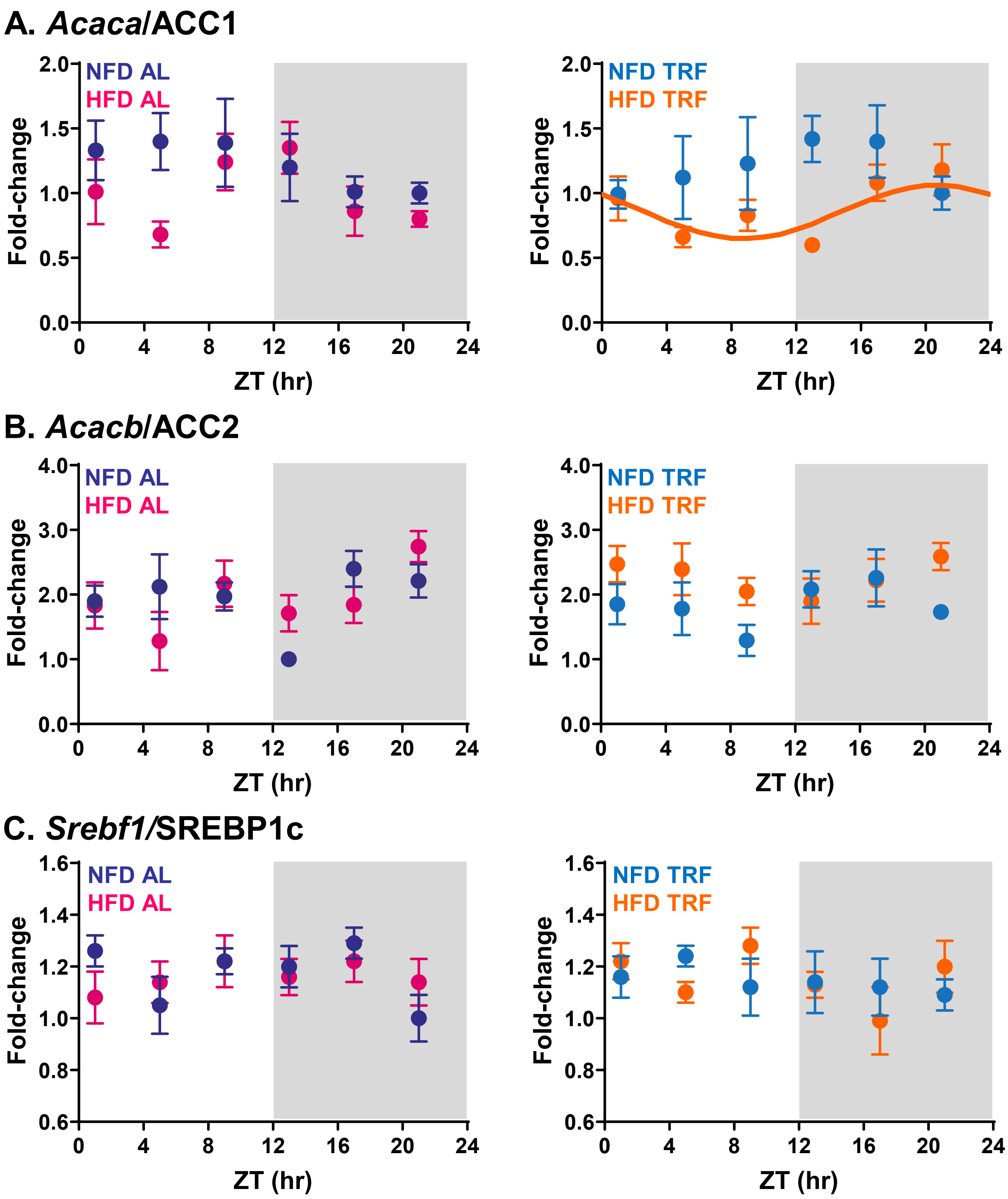

Supplemental Figure 2. Effect of high fat diet and time-restricted feeding on 24-h mRNA rhythms of lipid metabolism genes in the liver. Diurnal mRNA expression of **(A)** *Acaca*/ACC1, **(B)** *Acacb*/ACC2, and **(C)** *Srebf1*/SREBP-1c were measured in liver samples collected from NFD AL (dark blue), HFD AL (pink), NFD TRF (light blue), or HFD TRF (orange) mice at ZT1, 5, 9, 13, 17, and 21 (ZT0 – ZT12, lights on and ZT12 – ZT24 lights off/gray area). Results are presented as mean $\pm$ SEM for n = 4-6 mice per group at each ZT. Cosinor analysis was done using the nonlinear regression module in SPSS. A solid line indicates 24-h rhythmicity, and a significant cosine fit (R^2, $p \leq 0.05$), whereas the absence of line indicates arrhythmicity and a non-significant cosine fit (R^2, $p > 0.05$). Results from cosinor and ANOVA analyses are in **Suppl Tables 8** and **9**, respectively.

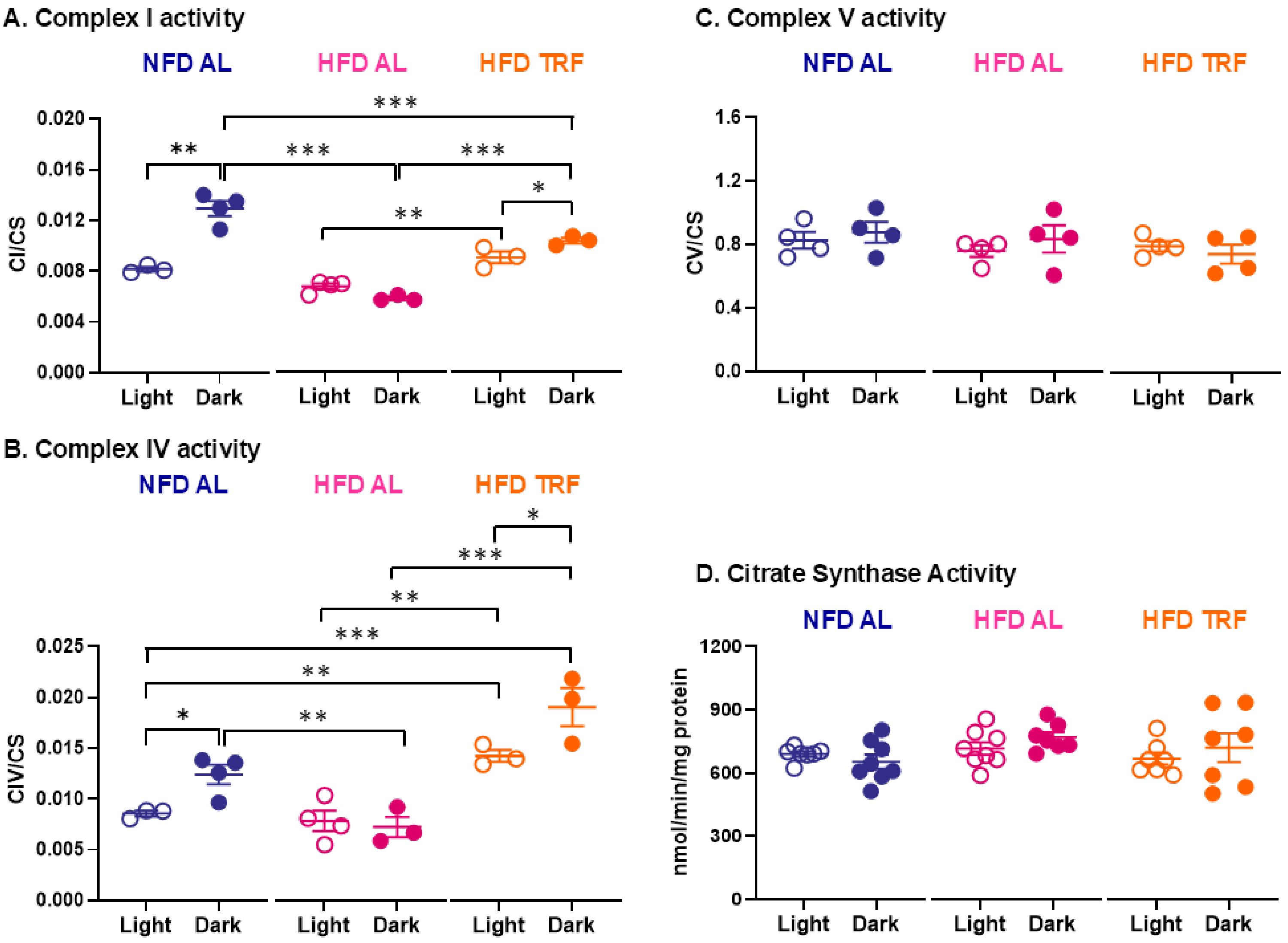

	Diet group		Time of day		Diet group × Time of day	
	F	p value	F	p value	F	p value
Complex I	72.47	< 0.001	30.61	< 0.001	30.06	< 0.001
Complex IV	38.98	< 0.001	10.42	0.006	4.04	0.041
Complex V	0.94	-	0.29	-	0.64	-
Citrate synthase	1.87	-	0.57	-	0.98	-

Supplemental Figure 3. Effect of high fat diet and time-restricted feeding on mitochondrial oxidative phosphorylation (OxPhos) complex and citrate synthase activities. Mitochondrial OxPhos complex and citrate synthase activities were determined in livers from NFD AL (dark blue), HFD AL (pink), and HFD TRF (orange) mice collected during the light (ZT1-ZT3) and dark (ZT13-ZT15) periods of the day. **(A)** Complex I, **(B)** Complex IV, and (**C**) Complex V activities were measured and normalized to (**D**) citrate synthase activity from the same animals. Citrate synthase activities are also included from additional animals in Fig 6. Results are presented as mean ± SEM and p-values from post-hoc tests are provided in graphs (*$p < 0.05$, **$p < 0.01$, ***$p < 0.001$) with ANOVA results provided in the table. F-values in bold font correspond to results with $p \leq 0.05$. A dash indicates $p > 0.05$.

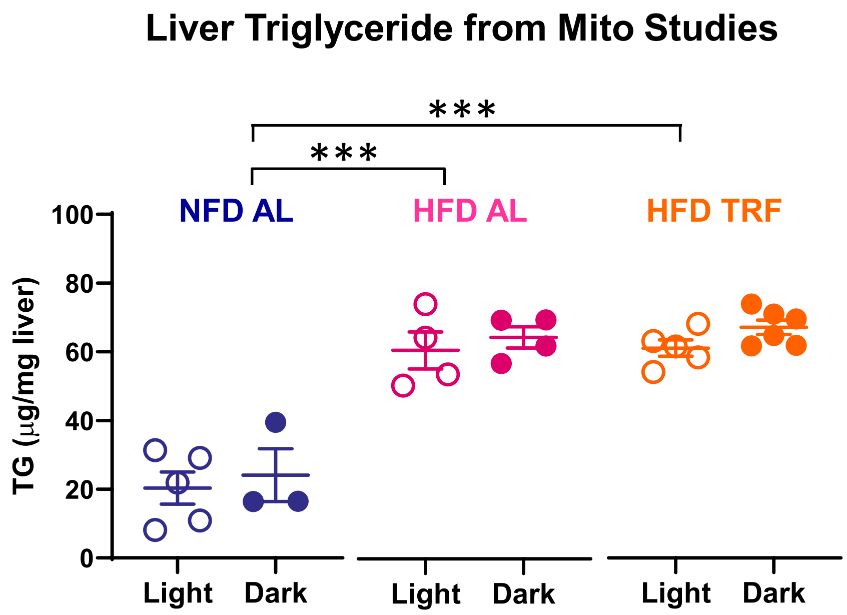

	F	p value
Diet group	64.44	< 0.001
Time of day	1.86	n.s.
Interaction	0.06	n.s.

Supplemental Figure 4. Effect of high fat diet and time-restricted feeding on liver triglyceride content. Liver triglyceride (TG) content was measured in samples collected from livers of NFD AL (dark blue), HFD AL (pink), and HFD TRF (orange) mice used in the mitochondrial studies (**Fig 6 and Suppl Fig 3**). Livers were collected during the light (ZT1-ZT3) and dark (ZT13-ZT15) periods of the day. Results are presented as mean $\pm$ SEM and p-values from post-hoc tests are provided in graphs (***$p < 0.001$) with ANOVA results provided in the table. F-values in bold font correspond to results with $p \leq 0.05$. A dash indicates $p > 0.05$.
